# Structural and functional coupling alterations in autism spectrum disorder with and without comorbid attention deficit hyperactivity disorder

**DOI:** 10.3389/fpsyt.2025.1704170

**Published:** 2026-01-15

**Authors:** Xiaolin Zhang, Yan Zhou, Luming Hu, Jingwen Yan, Xuntao Yin

**Affiliations:** 1 Department of Pediatric Rehabilitation, Guizhou Rehabilitation Hospital, Guiyang, China; 2 College of Engineering, Shantou University, Shantou, China; 3 Department of Radiology, Guangzhou Women and Children’s Medical Center, Guangzhou Medical University, Guangzhou, China; 4 Department of Psychology, School of Arts and Sciences, Beijing Normal University at Zhuhai, Zhuhai, China; 5 The School of Intelligent Manufacturing and Electrical Engineering, Guangzhou Institute of Science and Technology, Guangzhou, China

**Keywords:** autism spectrum disorder, attention deficit hyperactivity disorder, comorbidity, structural–functional coupling, resting-state fMRI, T1-weighted MRI

## Abstract

**Background:**

Autism spectrum disorder (ASD) and attention deficit hyperactivity disorder (ADHD) are highly comorbid. The neural basis of this comorbidity remains unclear. We compared brain structural-functional coupling (SC-FC coupling) across ASD subgroups and typically developing (TD) controls to parse the neurobiological heterogeneity of ASD.

**Methods:**

We analyzed T1-weighted and resting-state fMRI data from 331 participants from ABIDE II (130 ASD [39 ASD+ADHD, 91 ASD-only] and 201 TD). For each participant, we extracted multivariate structural features from T1-weighted images to construct an individual structural covariance network. SC-FC coupling for each brain region was quantified by correlating its observed functional connectivity profile with the profile predicted from individual structural features via linear regression.

**Results:**

Compared to TD individuals, the ASD group showed altered SC-FC coupling in networks critical for social cognition, emotion, sensory processing, and cognitive control: the default mode network (DMN), limbic system (LimN), somatomotor network (SMN), and frontoparietal network (FPN). Crucially, distinct patterns emerged between ASD subgroups. The ASD-only group had stronger coupling in the left inferior temporal gyrus (ITG.L). The ASD+ADHD group showed increased coupling in specific cerebellar regions: the right cerebellar lobule IX (Cerebellum_9_R) and right cerebellum Crus II (Cerebellum_Crus2_R).

**Conclusions:**

Our findings demonstrate both shared and subtype-specific alterations in SC-FC coupling in ASD. Comparing ASD subgroups clarifies that comorbid ADHD is associated with unique neural pathways, particularly involving cerebellar integration for attentional processes. Measuring SC-FC coupling offers a valuable approach for disentangling the heterogeneity in ASD and may aid in developing targeted interventions.

## Introduction

ASD and ADHD are two common neurodevelopmental disorders. They frequently co-occur, with 25.7–28% of individuals with ASD also meeting criteria for ADHD (1). Compared to individuals with ASD-only, ASD+ADHD show more severe impairments in sensory processing, language, and social interaction (2–6). Understanding the neural differences between these two groups is critical for advancing diagnosis and intervention.

Rs-fMRI is widely used to investigate brain function in ASD and ADHD. Studies apply various methods such as amplitude of low-frequency fluctuation (ALFF), regional homogeneity (ReHo), independent component analysis (ICA), and graph theory (7–12), including centrality measures (13). These studies reveal abnormalities in multiple systems, including DMN, SMN, visual areas, and reward circuits (14–17). Recent work also focuses on comparing ASD-only and ASD+ADHD groups. For example, Di Martino et al. find increased degree centrality in the basal ganglia in ASD+ADHD, while both groups show increased connectivity in temporal-limbic regions involved in social cognition (18).

However, existing research has limitations. Most studies rely only on rs-fMRI and overlook the contribution of brain structure to functional organization (15, 19). In addition, single-modality approaches often fail to capture the complex interactions between brain anatomy and function, such as the role of innate limbic circuitry (20).

To address these gaps, this study adopts a structural and functional coupling framework. This method combines T1-weighted MRI and rs-fMRI to assess the alignment between anatomical and functional networks. This approach allows us to leverage the rich anatomical information in T1-weighted images, focusing on multivariate tissue properties to construct the structural network, which provides a different yet complementary perspective to diffusion-based measures. Specifically, we use the approach described in (21), which constructs a structural connectivity matrix based on cortical thickness and a functional connectivity matrix from rs-fMRI. Coupling is then computed as the correlation between the two matrices. This approach captures the consistency between structural and functional connectivity, and recent studies show that it reflects disease-related brain changes with individual-level interpretability (21, 22).

This study aimed to compare SC-FC coupling patterns between ASD-only and ASD+ADHD individuals. Our analysis had two main steps. First, we identified core SC-FC alterations in ASD by comparing all ASD participants against TD controls. Second, we examined how ADHD comorbidity modulates these ASD-related alterations. We hypothesized that ADHD comorbidity does not necessarily introduce entirely novel abnormalities but rather modifies the expression of core ASD-related SC-FC coupling patterns in key brain circuits. Based on previous findings (1, 15), we hypothesize that the ASD+ADHD group will show altered coupling in limbic, reward, and visual networks. By integrating T1-weighted MRI and rs-fMRI data, our multimodal framework provides new insights into the brain mechanisms of ASD+ADHD and may help identify potential imaging biomarkers for clinical diagnosis.

Furthermore, to probe the multidimensional neurobiological sources underlying the observed SC-FC coupling variations, we employed principal component analysis (PCA). This data-driven approach allowed us to distill the high-dimensional structural connectivity matrices into a set of interpretable, brain-wide covariance patterns, and to investigate their expression across diagnostic groups.

## Materials and methods

**Figure 1:**
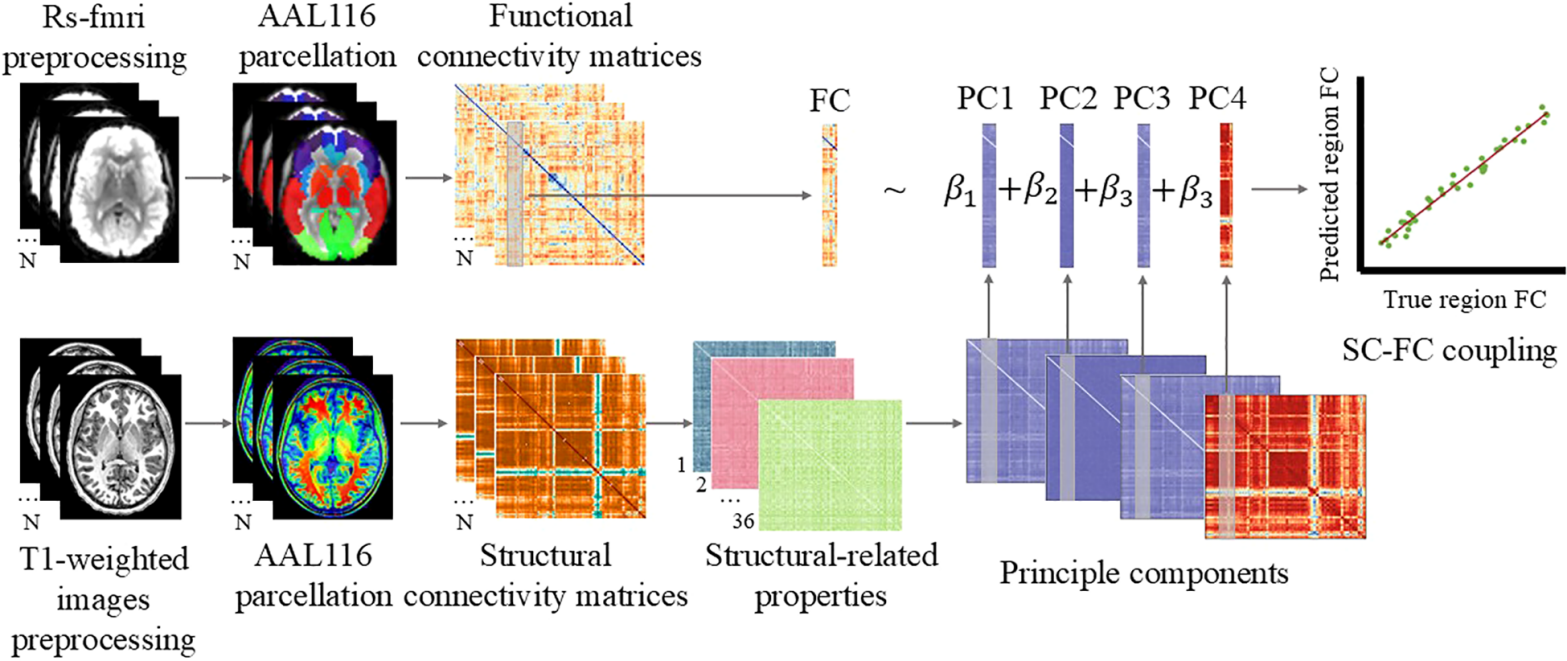
Overview of the Experimental Procedure. The process began with the preprocessing of rs-fMRI and T1-weighted MRI data. After whole-brain parcellation using the AAL116 atlas, functional and structural connectivity matrices were generated for each participant. Region-wise structural properties derived from these matrices underwent dimensionality reduction via principal component analysis. The resulting principal components were then used in a linear regression model to predict regional functional connectivity. Correlation coefficients between predicted and observed functional connectivity were computed to quantify structural and functional coupling across participants, revealing how structural features influence functional brain organization in ASD.

The overall workflow of this study was illustrated (**Figure 1**). We began by preprocessing rs-fMRI and T1-weighted MRI data. Following AAL116 brain parcellation, functional and structural connectivity matrices were generated. From the structural matrices, we derived a set of region-wise structural-related properties, which were further reduced using PCA. These principal components were then used in a linear regression model to predict region-level functional connectivity (FC), enabling the estimation of SC-FC coupling across participants. This pipeline allows us to investigate how structural features contribute to functional brain organization in ASD.

### Participants

**Figure 2:**
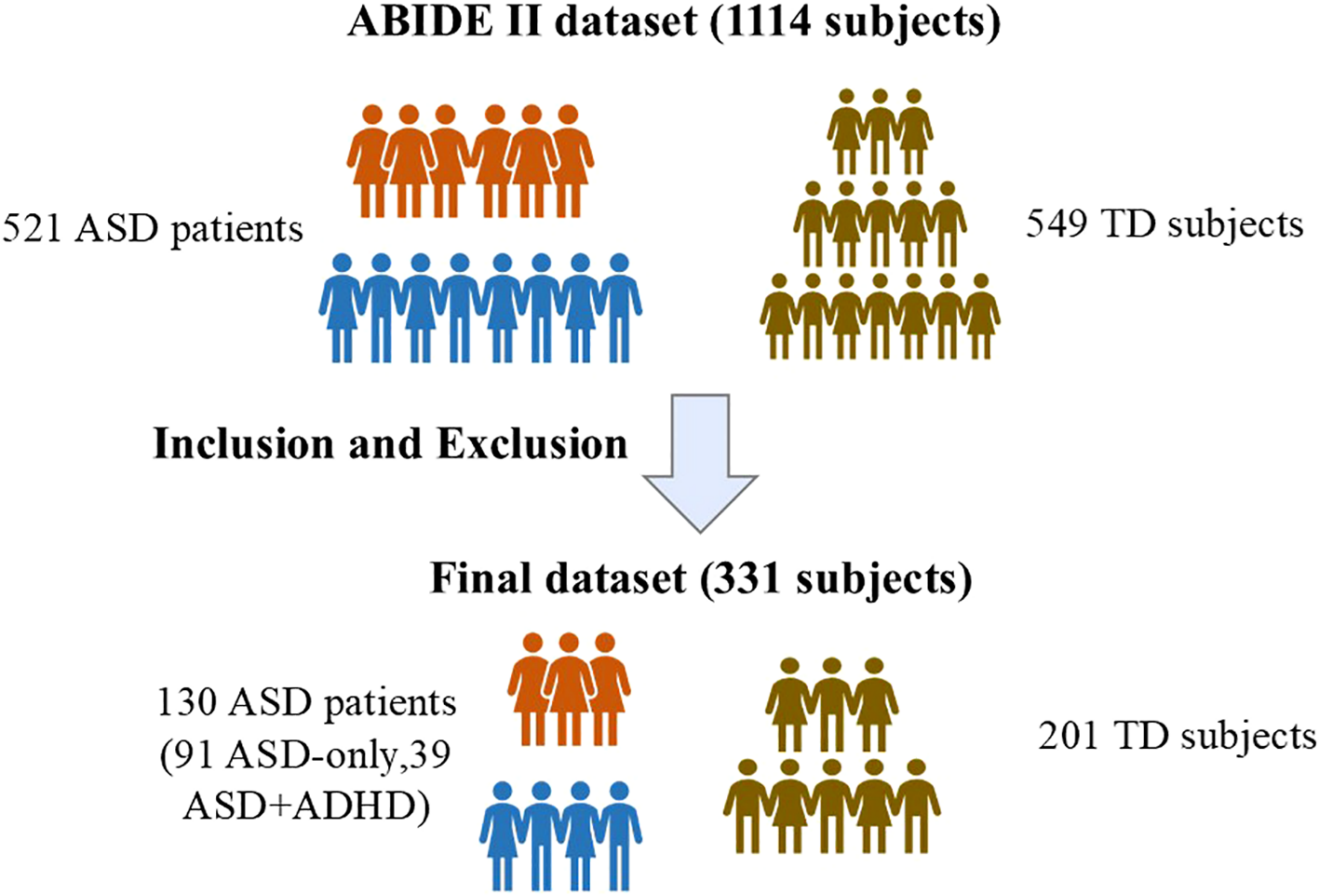
Participant Selection Flowchart from ABIDE II. First, individuals were categorized based on diagnostic labels: ASD or TD. Then, participants with mental health comorbidities other than ADHD were excluded. Finally, the remaining ASD participants were divided into two subgroups: ASD+ADH and ASD-only.

We utilized data from the Autism Brain Imaging Data Exchange II (ABIDE II, https://fcon_1000.projects.nitrc.org/indi/abide/abide_II.html), a publicly accessible database established to advance ASD research (23). This resource was selected over ABIDE I due to its enhanced characterization of comorbidities such as ADHD, which is crucial for subtyping analyses. The full dataset comprises 1,114 participants (521 with ASD and 593 TD individuals) from 19 sites. All diagnostic classifications (ASD, ASD+ADHD, TD) provided by ABIDE II were based on established diagnostic protocols at the participating sites. Our participant selection followed a standardized filtering procedure (17) (**Figure 2**) to derive the stratified ASD subgroups for this study. We included participants who had both whole-brain rs-fMRI and T1-weighted scans and exhibited a mean framewise displacement < 2.5 mm, while excluding those with comorbidities other than ADHD.

Beyond this initial selection, further inclusion criteria were applied to ensure data quality and comparability. The participant dataset had to include whole-brain coverage rs-fMRI scans and T1-weighted images. In addition, excessive head motion artifacts may increase the false positive rate, particularly in ADHD patients (23–25). Considering this situation, we selected participants from ABIDE II with a mean framewise displacement (mFD) of less than 2.5 mm. To ensure comparability between groups, age, gender, and performance IQ (PIQ) were matched between ASD and TD, as well as between ASD+ADHD and ASD-only. Furthermore, autism traits were assessed using two parent-reported scales, the social interaction subscale of the Autism Diagnostic Interview-Revised (ADI-R) (26) and the Social Responsiveness Scale (SRS) (27). Significantly, the ABIDE II dataset only provides diagnostic information for comorbid psychiatric conditions, lacking accompanying questionnaire data or symptom scores. Due to the variation in eye state among different sites, we also collected the participants’ eye state during rs-fMRI scans.

After meticulous data selection across 19 sites, only 8 sites ultimately contributed 331 participants matching the criteria. This group included 130 individuals with ASD, specifically 39 with ASD+ADHD, 91 with ASD-only, and 201 TD individuals. ADHD subtypes among ASD patients and their medication status are detailed (**Table 1**).

**Table 1:** Demographic and clinical characteristics.

Characteristics	ASD+ADHD (*n* =39)	ASD-only (*n* = 91)	*p*	Autism (*n* = 130)	TD (*n* = 201)	*p*
Age	11.24 ± 5.46 [5.60 - 26.62]	13.61 ± 6.58 [5.13 - 34.76]	0.059^a^	12.90 ± 6.34 [5.13 - 34.76]	12.25 ± 6.35 [5.89 - 46.6]	0.975^a^
Performance IQ	104.00 ± 19.42 [61 - 147]	102.96 ± 19.10 [64 - 149]	0.779^b^	103.30 ± 18.54 [61 - 149]	111.55 ± 12.17 [79 - 147]	<0.001^a^
Sex(male/female)	34/5	81/10	0.765^c^	115/15	136/15	<0.001^c^
ADI_R_social (*N*_ASD+ADHD_ = 32; *N*_ASD-only_ = 32)	18.84 ± 5.50 [3.0 - 28.0]	16.81 ± 6.28 [6.0 - 27.0]	0.174^b^	17.83 ± 5.94 [3.0 - 28.0]		
SRS_Total_T (*N*_ASD+ADHD_ = 21; *N*_ASD-only_ = 61; *N*_TD_ = 149)	81.62 ± 23.39 [48.0 - 141.0]	71.61 ± 19.23 [25.0 - 114.0]	0.09^a^	74.17 ± 20.70 [25.0 - 141.0]	40.64 ± 11.21 [4.0 - 65.0]	<0.001^a^
Mean FD	0.14 ± 0.13 [0.04 - 0.65]	0.09 ± 0.05 [0.03 - 0.27]	0.05^a^	0.11 ± 0.08 [0.03 - 0.65]	0.10 ± 0.06 [0.02 - 0.34]	0.201^a^
Eye status(open/closed)	35/4	76/15	0.36^c^	111/19	176/25	0.686^c^

Data are presented as mean ± SD [range].

ASD, autism spectrum disorder; ADHD, attention deficit hyperactivity disorder; TD, typical developmental; ASD + ADHD, the comorbidity of ASD and ADHD; ASD-only, ASD without ADHD; ADI_R_social, social interaction subscale of Autism Diagnostic Interview-Revised; SRS_Total_T, Social Responsiveness Scale Total Score; FD, Framewise displacement.

a Factor represents Mann–Whitney test.

b factor indicates two-sample t test.

c factor represent Chi-square test. P-value is calculated by these three tests. Participants who satisfied the inclusion criteria are from eight sites in ABIDE II, EMC_1, IP_1, KKI_1, KUL_3, NYU_1, NYU_2, SU_2 and UCD_1.

Data presented compares 39 participants with ASD+ADHD, 91 participants with ASD-only, 130 participants in the combined autism group, and 201 typically developing participants. Measures include age ranges, performance IQ scores, male to female ratios, ADI-R social domain scores with available data for 32 ASD+ADHD and 32 ASD-only cases, SRS total raw scores with available data for 21 ASD+ADHD, 61 ASD-only, and 149 TD cases, mean framewise displacement values, and eye status during assessment.

### Image acquisition and preprocessing

The image datasets were acquired on 3T scanners. The sequence parameters for each site and scanner types are summarized in the previously introduced ABIDE II study (28). All available rs-fMRI and T1-weighted images were preprocessed using RESTplus v1.30.

### Functional MRI preprocessing

The preprocessing of rs-fMRI data included the following steps: (1) removal of the first ten volumes to allow for magnetic field stabilization; (2) slice timing correction; (3) head motion correction using a six-parameter rigid body transformation; (4) co-registration to the individual’s T1-weighted image and spatial normalization to the Montreal Neurological Institute (MNI) standard space with 3-mm isotropic voxels using the Diffeomorphic Anatomical Registration Through Exponentiated Lie Algebra (DARTEL) tool; (5) linear detrending and bandpass filtering (0.01–0.08 Hz) to retain low-frequency fluctuations; and (6) regression of nuisance covariates, including Friston’s 24-parameter model of head motion, and signals from white matter and cerebrospinal fluid. Global signal regression was not applied, consistent with prevailing controversies in the field (29, 30).

### Structural MRI (T1-weighted) preprocessing

The T1-weighted images underwent standard preprocessing to ensure data quality and compatibility for spatial analysis and subsequent feature extraction. The pipeline consisted of: (1) intensity non-uniformity (bias) correction; (2) skull stripping to remove non-brain tissue; (3) spatial normalization to the MNI standard space using the high-dimensional DARTEL registration; and (4) resampling to a uniform 1×1×1 mm³ isotropic voxel size.

### Network construction

#### Functional brain network construction

Based on Automated Anatomical Labeling (AAL) atlas template (31), we divided the human brain into 116 regions and built a functional brain network for each participant using preprocessed rs-fMRI images. In the network, each node represents a brain region, and the edges between nodes represent the functional connections between regions. To construct this network, we first extracted the time series for each region and calculated the Pearson correlation coefficient between the time series of each pair of regions. The correlation coefficient reflects the strength of FC between regions, resulting in a 116×116 connection matrix. To make the data distribution more uniform and facilitate subsequent statistical analysis, we converted the Pearson correlation coefficients to Fisher’s *z* values.

#### Structural feature extraction and radiomics network construction

The structural brain network was derived from T1-weighted images using a radiomics similarity approach. The feature extraction and selection process began with the standardized extraction of 47 radiomics features from each of the 116 AAL regions in the preprocessed T1-weighted images. This set included 14 first-order (intensity-based) features and 33 second-order texture features, as defined by Aerts et al (32). To eliminate redundancy from highly correlated features, we computed the pairwise Pearson correlation between all 47 features across all regions and participants. Feature pairs with a correlation coefficient greater than 0.9 were considered redundant; in such cases, one feature was randomly removed from the pair. This procedure yielded a final set of 25 non-redundant features. Consequently, for each individual, the radiomics data were organized into a matrix of dimensions 25 (selected features) × 116 (brain regions).

Subsequently, the radiomics similarity network (R2SN) was constructed. This was achieved by calculating the Pearson correlation coefficient between the 25-dimensional radiomics feature vectors of every pair of brain regions, resulting in a 116×116 structural similarity matrix for each participant (33). Thus, each element in this matrix represents the similarity of multivariate tissue properties between two regions, defining the edge weight in the radiomics-based structural network.

Finally, to characterize the network’s topology, we computed a comprehensive set of graph-theoretical metrics. In total, 34 distinct metrics were calculated, capturing properties across eight key domains: centrality measures (13), efficiency and connectivity, structure and modularity (34, 35), distance and path, density and entropy, subgraph composition (35), and heterogeneity. The calculation of these metrics was based on established network analysis frameworks (36). For example, we examined centrality (e.g., degree, betweenness), integration (e.g., global efficiency), and segregation (e.g., clustering coefficient). In addition, the Euclidean distance between the centroids of every pair of AAL regions was computed as a spatial predictor.

### Principal component analysis of structural features

We used principal component analysis (PCA) to reduce the dimensionality of the structural features and extract dominant patterns. For each participant, a high-dimensional structural feature vector was constructed by concatenating the vectorized upper triangle of the 116×116 structural connectivity (SC) matrix, the 34 graph-theoretical metrics, and the vectorized Euclidean distance matrix. All features were z-score normalized before PCA. We retained the top four principal components (PCs), which collectively accounted for over 80% of the total variance. To interpret the biological meaning of these PCs, we examined the component loadings to determine which original features contributed most to each component.

To interpret the biological meaning of these data-driven components, we examined their loadings onto the original features. This *post-hoc* analysis revealed that each PC was predominantly driven by a distinct set of graph-theoretical or spatial features. We therefore interpreted the four PCs as representing fundamental ‘brain network organizational dimensions,’ such as global efficiency or spatial constraint. This interpretation was crucial as it allowed us to understand what these components represented before using them as predictors in the SC-FC coupling linear regression model. Additionally, the component scores for these PCs were independently used in between-group comparisons to identify diagnosis-specific alterations in macroscopic structural covariance patterns.

### Regional SC-FC coupling

To model the structural function coupling, we employed a region-level multiple linear regression framework. For each brain region, its entire functional connectivity (FC) profile, which was represented as a vector from the 116×116 FC matrix, served as the dependent variable. The top four principal components (PCs), which were previously derived from the structural feature set and accounted for over 80% of the total variance, were used as independent variables to predict the FC profile. Prior to model fitting, all regional FC vectors were z-score normalized to mitigate the influence of differences in scale and distribution.

After fitting the regression model for a given region, we quantified the SC-FC coupling strength for that region by calculating the Pearson correlation coefficient between the predicted FC vector and the empirically observed FC vector. This coefficient, which ranges from -1 to 1, reflects the degree to which the multivariate structural patterns can predict the functional connectivity of the region, with higher values indicating stronger structural constraints on function.

In addition to this region-specific measure, we computed a global SC-FC coupling value for each participant. This was achieved by first assembling the predicted FC vectors from all regions into a single predicted whole-brain FC matrix, and then calculating the Pearson correlation between this predicted matrix and the actual observed FC matrix.

### Statistical analysis

All statistical analyses were performed using in-house scripts in Python (v3.9) with the Scipy and Statsmodels libraries. Following previous large-scale neuroimaging studies on ASD and ADHD (17, 24, 37), we included age, sex, performance IQ (PIQ), mean framewise displacement (mFD), total intracranial volume (TIV), eye status during scanning, and acquisition site as covariates of no interest in all statistical models to reduce confounding effects (38, 39). All statistical tests were corrected for multiple comparisons using the Benjamini-Hochberg false discovery rate (FDR) method. A two-tailed p-value < 0.05 was considered statistically significant.

### Analytical rationale overview

Our statistical analysis followed a two-stage procedure designed to address distinct but complementary research questions. The first stage (ASD vs. TD) aimed to identify the core set of brain regions exhibiting significant SC-FC coupling alterations in the ASD population as a whole, thereby establishing the foundational “ASD-related neural signature.” The second stage (ASD-only vs. ASD+ADHD) was conducted specifically within the brain regions identified as abnormal in the first stage. This focused approach directly tests our hypothesis that ADHD comorbidity exerts a modulatory effect on the core ASD-related SC-FC signature, rather than acting through entirely independent neural pathways. This hierarchical design enhances the biological interpretability of subgroup differences by tethering them to the established neuropath physiology of ASD.

### Identification of ASD-related SC-FC coupling alterations

To identify the core SC-FC coupling alterations associated with ASD, we performed two-sample t-tests on the regional SC-FC coupling strength between all ASD participants (n=130) and the TD control group (n=201) (40). Brain regions showing significant differences after FDR correction were considered to exhibit abnormal SC-FC coupling in ASD and were defined as the “ASD-signature regions” for all subsequent subgroup analyses. To facilitate visualization and interpretation, all 116 brain regions defined by the AAL atlas were categorized into nine canonical functional networks: Auditory (AudN), Dorsal Attention (DAN), Default Mode (DMN), Frontoparietal (FPN), Limbic (LimN), Somatomotor (SMN), Subcortical (SUB), Ventral Attention (VAN), and Visual (VIS) (Additional file 1: **Supplementary Table S1**). This network assignment was based on prior mappings of AAL regions to functional systems in the literature (41). Cohen’s *d* was calculated to assess the effect size of group differences in each region.

### Considerations for statistical independence and power and subgroup comparison

When comparing the ASD-only and ASD+ADHD subgroups within the pre-defined ASD-signature regions, we considered two methodological issues: statistical non-independence and the lower statistical power from the smaller ASD+ADHD group (42). We used two strategies to ensure our findings were robust.

First, to check if the subgroup differences were independent of our initial analysis, we looked at how the key regions behaved in our other comparisons (ASD-only vs. TD and ADHD vs. TD). We reasoned that if a region was a strong ASD marker but showed a different pattern in the subgroups, this meant ADHD changed the core ASD feature. If a region was a weak ASD marker but a strong ADHD marker and differed between subgroups, it meant the comorbid group had a distinct ADHD feature. This cross-comparison helps show that the differences are real biological effects and not just statistical artifacts (43).

Second, because the ASD+ADHD group was smaller (n=39), we focused on effect sizes, specifically Cohen’s *d* and its 95% confidence interval (CI), in addition to p-values (44, 45). This helps show that a finding is biologically meaningful even with unequal group sizes (46).

Following this approach, we used two-sample t-tests to compare SC-FC coupling between the ASD-only (n=91) and ASD+ADHD (n=39) subgroups within the ASD-signature regions, including the same covariates. For significant regions, we reported Cohen’s *d* and 95% CI.

### Correlation with clinical measures

To explore the clinical relevance of the observed SC-FC coupling alterations, we performed partial correlation analyses between the coupling strength in significant regions and autism symptom severity scores (i.e., ADI-R social subscale or SRS scores). These analyses were conducted within the ASD group and were adjusted for the same set of covariates (age, sex, PIQ, mFD, TIV, eye status, and site).

### Demographic and clinical data analysis

For demographic and clinical variables, differences in continuous variables between groups were analyzed using the Mann–Whitney U test for non-normally distributed data and the two-sample t-test for normally distributed data. Categorical variables (i.e., sex and eye status) were tested using the Chi-square test. A p-value < 0.05 was considered significant for these demographic comparisons.

## Results

### Demographic and clinical characteristics

**Table 1** presents the demographic and clinical data. In terms of demographic features, there were no significant differences in age (*p* = 0.059), gender (*p* = 0.765), or Performance IQ (PIQ) (*p* = 0.779) between the ASD+ADHD and ASD-only groups. The groups were well-matched on these variables. Similarly, there was no significant difference in age between the ASD and TD groups (*p* = 0.975). However, the gender differed significantly between the two groups (*p* < 0.001), and the PIQ was significantly higher in the TD group compared to the ASD group (*p* < 0.001).

Regarding motion artifacts, the ASD+ADHD group showed a slightly higher mean frame displacement (mean FD) than the ASD-only group, with a trend toward significance (*p* = 0.05). There was no significant difference in mean FD between the ASD and TD groups (*p* = 0.201). There were no significant differences in eye status (open/closed) in either comparison.

In clinical characteristics, the SRS total T-score was significantly higher in the ASD group compared to the TD group (*p* < 0.001), indicating more pronounced social difficulties in the ASD group. Within the ASD subgroup, the SRS_T score was higher in the ASD+ADHD group compared to the ASD-only group, but this difference did not reach statistical significance (*p* = 0.09). There was also no significant difference in the ADI-R social score between the two ASD subgroups (*p* = 0.174).

### Aberrant SC-FC coupling between participants with ASD and typical developmental

**Figure 3:**
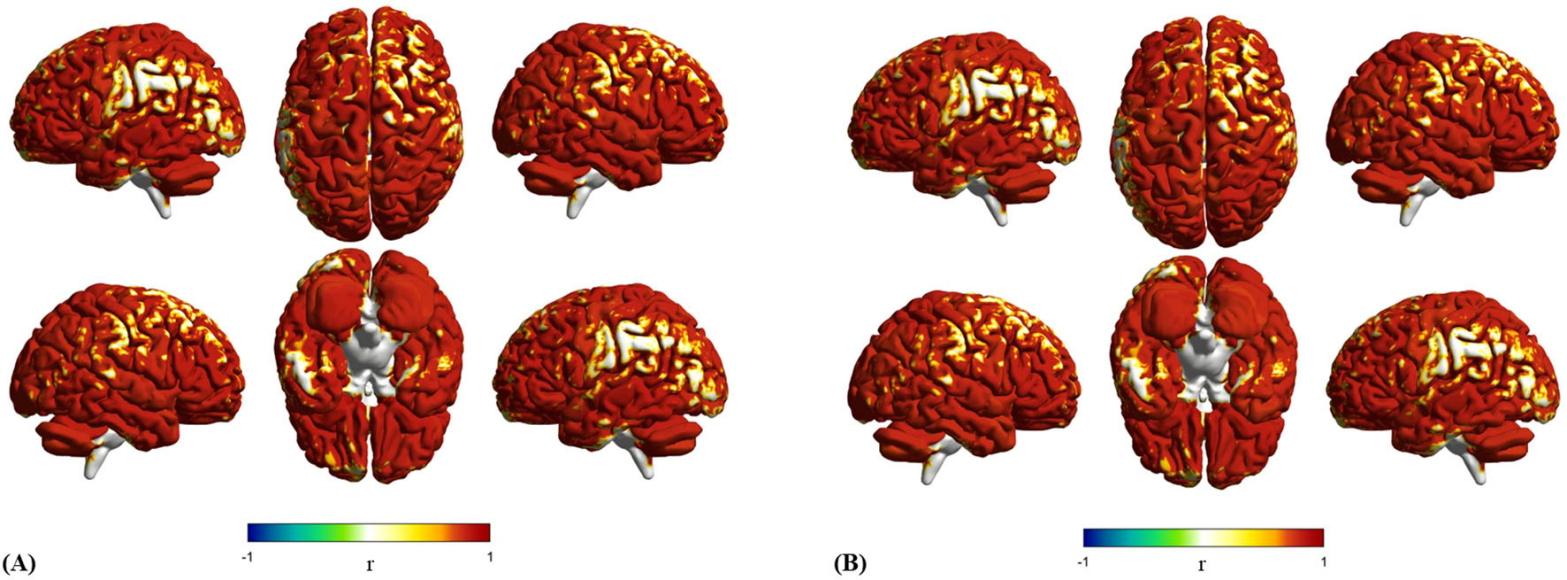
Mean SC-FC Coupling Across TD and ASD. TD shows consistent coupling within the default mode network, with peak connectivity in the thalamus. In contrast, ASD reveals shifted patterns where auditory regions show strongest coupling while cerebellar areas exhibit the weakest connections. **(a)** Mean SC-FC coupling of TD. **(b)** Mean SC-FC coupling of ASD.

Structural connectivity showed a strong link to functional connectivity in TD individuals (mean [SD] SC-FC coupling, 0.801 [0.07]). This means brain structure helps shape how brain regions work together. But this link differed across brain area (**Figure 3a**). The maximum mean SC-FC coupling was located at the left thalamus is 0.873 [0.0455], and the minimum mean SC-FC coupling located at the left precuneus is 0.749 [0.101], both regions within the DMN (44). This regional variability reflects differences in the extent to which structural connectivity supports functional dynamics.

Individuals with ASD, including those with ASD-only and ASD+ADHD, also showed SC-FC coupling (mean [SD] SC-FC coupling, 0.7944 [0.0819]). This global coupling was slightly lower than in TD. However, a distinct spatial pattern of regional coupling compared to TD was observed (**Figure 3b**). In ASD, the right Heschl’s gyrus (HES.R) within the AudN showed the strongest coupling at 0.8597 [0.0469]. Vermis_10 in the SMN network exhibited the weakest coupling at 0.7259 [0.1340], demonstrating higher variability than the weakest region in TD. This change in where the strongest and weakest coupling occur suggested altered structure-function relationships in ASD.

**Figure 4:**
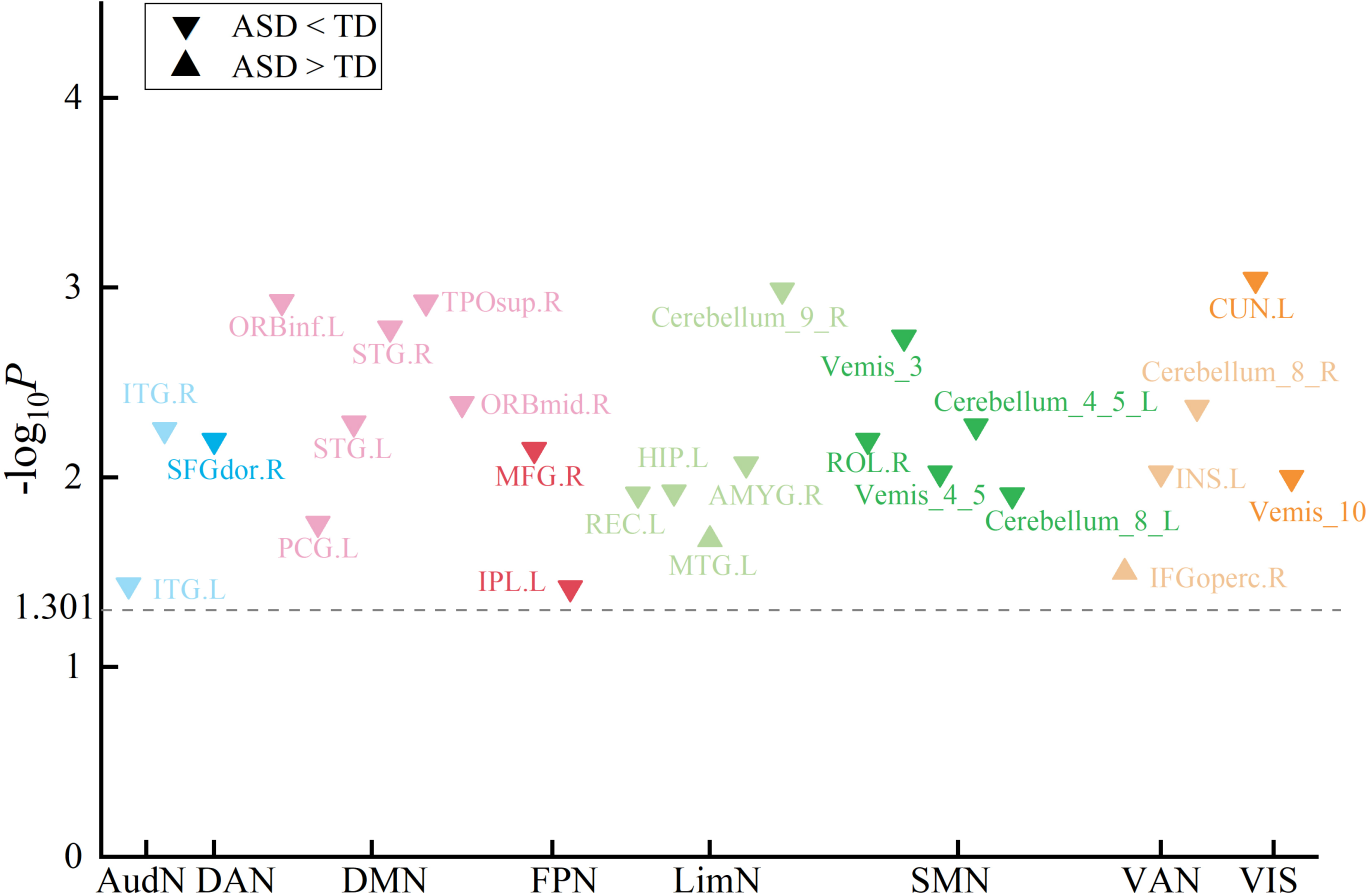
Manhattan plot of SC-FC coupling differences between ASD and TD. Brain regions are grouped according to eight canonical functional networks: AudN (Auditory Network), DAN (Dorsal Attention Network), DMN (Default Mode Network), FPN (Frontoparietal Network), LimN (Limbic Network), SMN (Somatomotor Network), VAN (Ventral Attention Network), and VIS (Visual Network).

**Figure 5:**
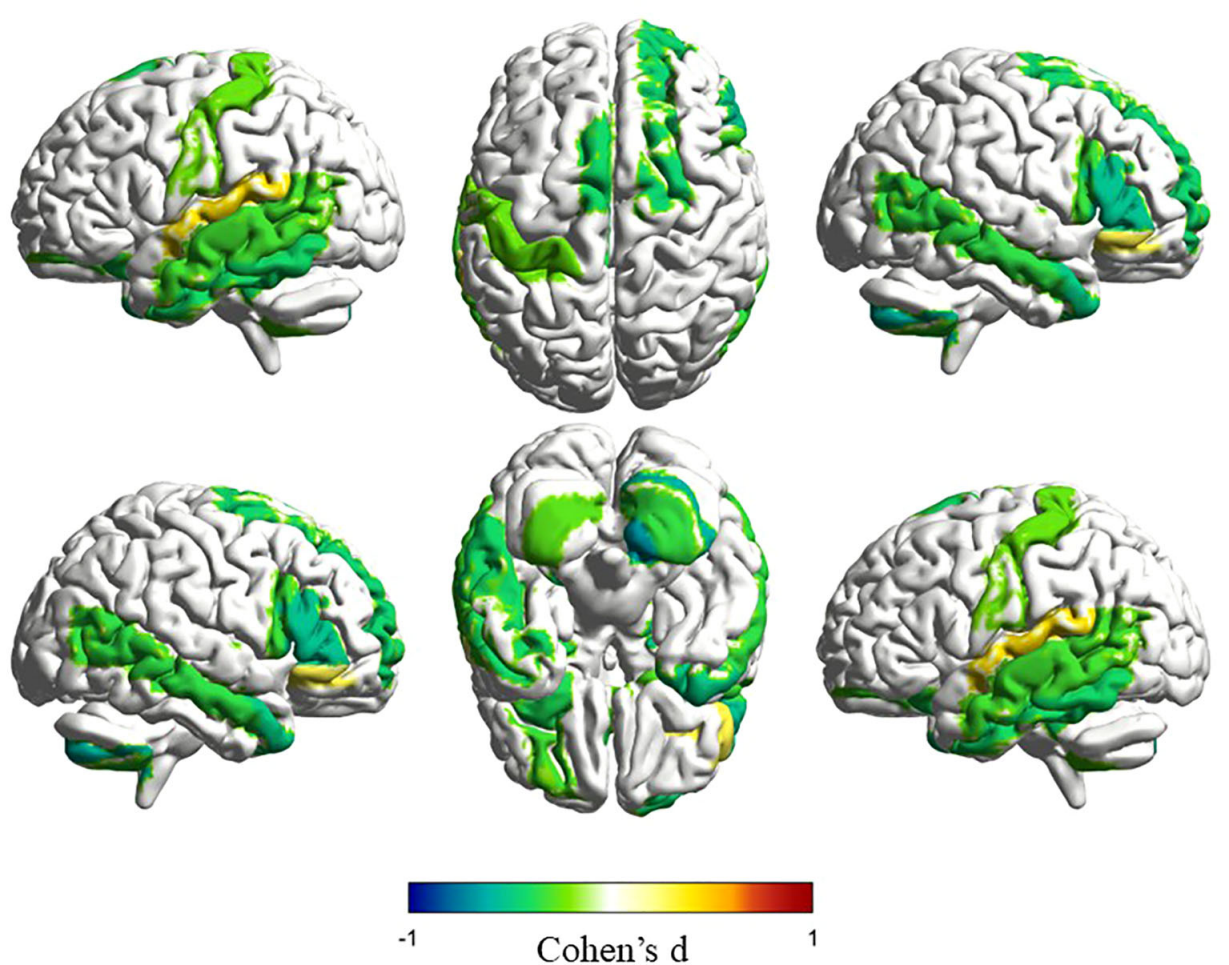
Effect size of SC-FC coupling differences between ASD and TD measured by Cohen’s *d*. Cohen’s *d* values reveal asymmetric reductions across networks: 24 regions show decreased coupling in ASD versus only 2 increases. Pronounced decreases emerge in cerebellar and limbic regions while frontoparietal and ventral attention networks show bidirectional changes.

Consistent with this altered spatial organization, we identified significant group differences in SC-FC coupling across 26 brain regions after FDR correction (*p* < 0.05) (**Table 2**), with spatial visualization (**Figure 4**). Among these regions, 24 showed reduced coupling in ASD compared to TD controls, while only 2 showed increased coupling. Effect sizes quantified via Cohen’s *d* (**Figure 5**) and revealed distinct network-level patterns: Five DMN regions exhibited reduced coupling with moderate-to-strong effect sizes, showing Cohen’s *d* values spanning -0.322 to -0.533. Similarly, five LimN regions demonstrated reductions with d values from -0.317 to -0.677. The SMN network displayed consistent decreases across five cerebellar regions at *d* = -0.270 to -0.431. Conversely, FPN and VAN networks featured bidirectional coupling alterations. Overall effect sizes confirmed moderate-to-strong impairments throughout affected regions, with particularly pronounced reductions in cerebellar and limbic areas (**Figure 5**). These findings suggest network-specific reorganization of structural and functional coupling in ASD.

**Table 2:** Statistical analysis of differences between ASD and TD. ASD and TD groups show differences in structural and functional coupling across brain regions.

ID	Name	Network	*t*	Cohen’s *d*	95%CI	*p_adj_*
89	ITG.L	AudN	-4.122	-0.313	[-0.563, -0.063]	0.037
90	ITG.R	AudN	-7.677	-0.350	[-0.600,-0.099]	0.006
4	SFGdor.R	DAN	-7.428	-0.443	[-0.694, -0.192]	0.006
10	ORBmid.R	DMN	-8.396	-0.533	[-0.881, -0.184]	0.004
15	ORBinf.L	DMN	-10.732	-0.420	[-0.671, -0.169]	0.001
35	PCG.L	DMN	-5.478	-0.369	[-0.620, -0.119]	0.018
81	STG.L	DMN	-7.860	-0.393	[-0.643, -0.142]	0.005
82	STG.R	DMN	-10.084	-0.404	[-0.655, -0153]	0.002
84	TPOsup.R	DMN	-10.705	-0.322	[-0.572, -0.007]	0.001
8	MFG.R	FPN	-7.189	-0.329	[-0.579, -0.079]	0.007
61	IPL.L	FPN	-4.046	-0.250	[-0.500, -0.0008]	0.038
27	REC.L	LimN	-6.145	-0.317	[-0.567, -0.067]	0.012
37	HIP.L	LimN	-6.205	-0.373	[-0.624, -0.128]	0.012
42	AMYG.R	LimN	-7.012	-0.522	[-0.870, -0.174]	0.009
85	MTG.L	LimN	5.318	0.427	[0.080, 0.774]	0.021
110	Cerebellum_9_R	LimN	-11.049	-0.677	[-1.027, -0.327]	0.001
18	ROL.R	SMN	-7.412	-0.292	[-0.542, -0.042]	0.006
92	Vemis_3	SMN	-9.852	-0.412	[-0.662, -0.161]	0.005
93	Vemis_4_5	SMN	-6.639	-0.416	[-0.668, -0.164]	0.002
101	Cerebellum_4_5_L	SMN	-7.779	-0.431	[-0.683, -179]	0.005
107	Cerebellum_8_L	SMN	-6.120	-0.270	[-0.519, -0.018]	0.012
12	IFGoperc.R	VAN	4.401	0.295	[0.045, 0.545]	0.032
29	INS.L	VAN	-6.651	-0.381	[-0.632, -0.131]	0.010
108	Cerebellum_8_R	VAN	-8.292	-0.341	[-0.592, -0.089]	0.005
45	CUN.L	VIS	-11.270	-0.384	[-0.634, -0.133]	0.001
98	Vemis_10	VIS	-6.690	-0.592	[-0.941, -0.243]	0.010

Significant SC-FC coupling variations between ASD and TD groups were identified in 26 regions after FDR correction (*p* < 0.05). Results include t-statistics effect sizes as Cohen’s *d* and adjusted p-values for each region.

### Disrupted SC-FC coupling in ASD subgroups

To further explore the differences in SC-FC coupling between clinical subtypes of ASD, we divided participants into ASD-only and ASD+ADHD groups. Significant group-level differences were observed in the ITG.L, the Cerebellum_9_R and Cerebellum_Crus2_R.

**Figure 6:**
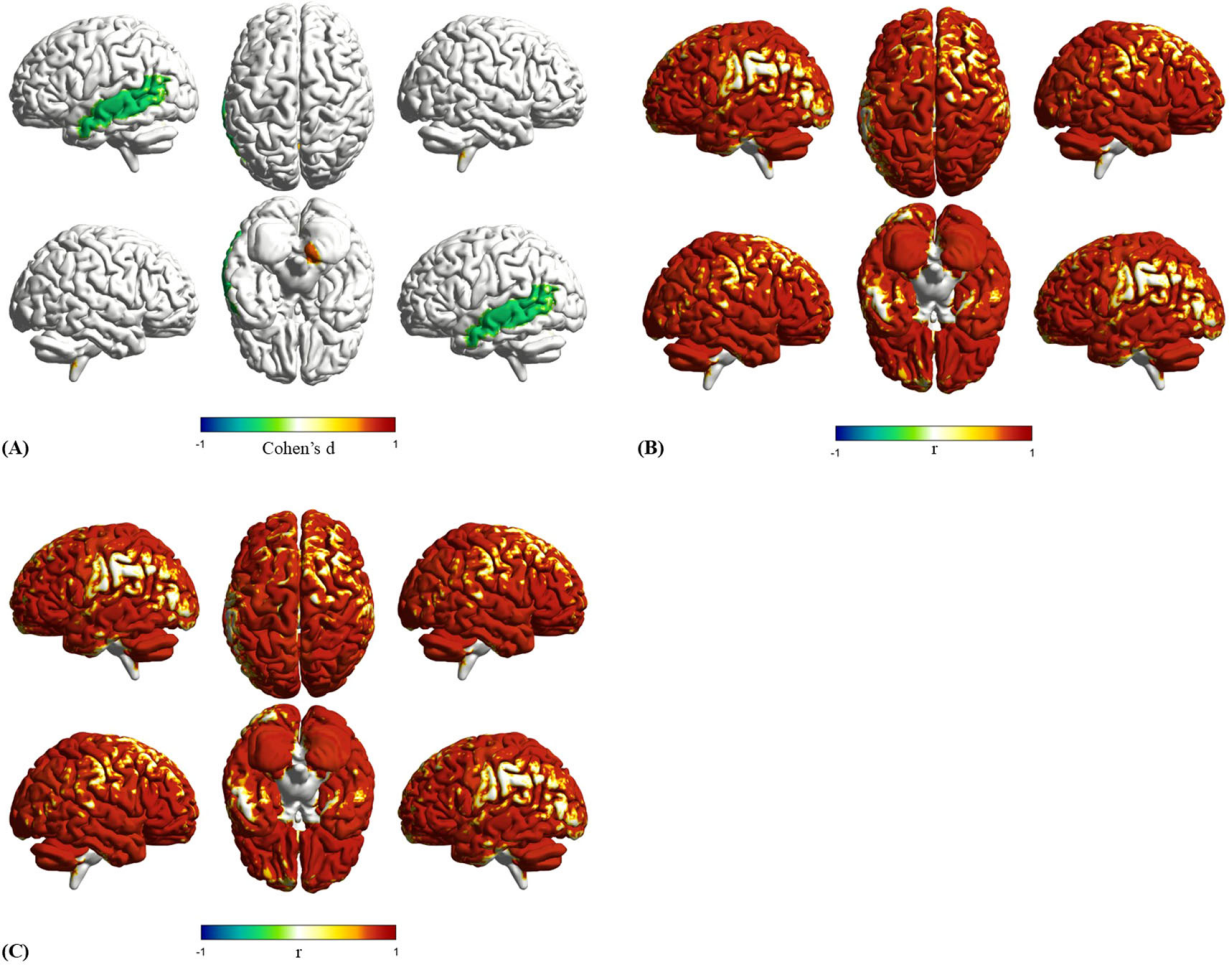
Group Differences in SC-FC Coupling Between ASD Subgroups. Comparative analysis illustrating differences in connection strength and spatial distribution of average SC-FC coupling for ASD-only versus ASD+ADHD. **(a)** Effect size of SC-FC coupling differences between ASD-only and ASD+ADHD measured by Cohen’s d. **(b)** Mean SC-FC coupling of ASD-only. **(c)** Mean SC-FC coupling of ASD+ADHD.

**Figure 7:**
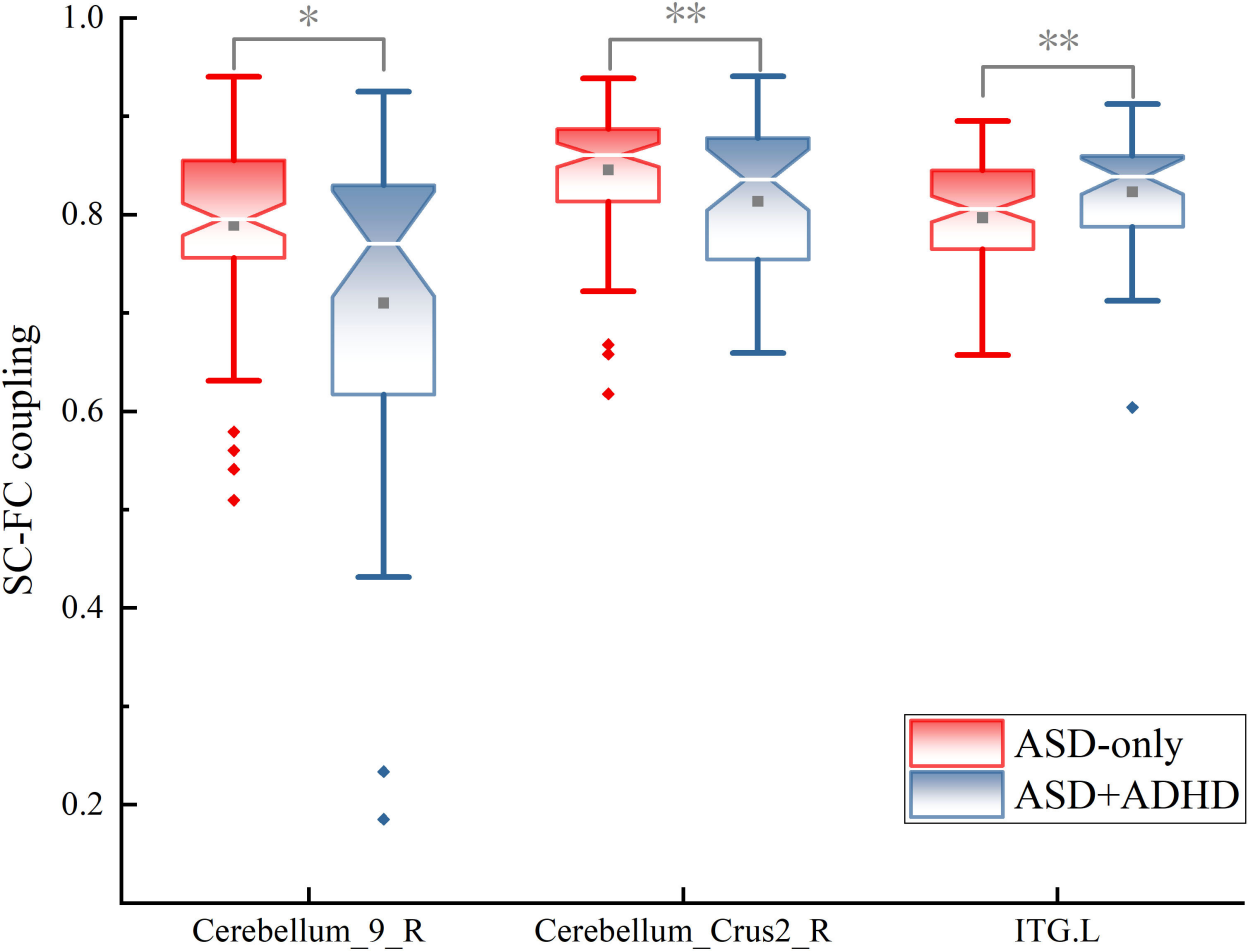
SC-FC coupling in significant regions for ASD-only and ASD+ADHD: Boxplots characterize differential SC-FC patterns in the left inferior temporal gyrus and cerebellar subregions where comorbidity modulates structural-functional integration (* p<0.05, ** p<0.01).

Group differences in SC-FC coupling in ASD subgroups were observed, with Cohen’s *d* quantifying these variations (**Figure 6a**). Mean coupling intensities are mapped separately for the ASD-only and ASD+ADHD (**Figures 6b, c**). Significantly stronger coupling occurred in the ASD-only group at ITG.L (Cohen’s *d* = -0.437, *p* < 0.05). Conversely, the ASD+ADHD showed enhanced coupling at Cerebellum_9_R (*d* = 0.681; *p* < 0.01) and Cerebellum_Crus2_R (*d* = 0.481; *p* < 0.01). This spatial pattern suggests ADHD comorbidity alters the integration of brain structure and function. Boxplots display group differences in coupling strength for these regions (**Figure 7**). Complete statistical details are provided (**Table 3**).

**Table 3:** Statistical results for brain regions with significant SC-FC coupling differences.

ID	Name	Network	*t*	Cohen’s *d*	95%CI	*p*_adj_
89	ITG.L	AudN	-6.729	-0.437	[-0.819, -0.055]	0.010
110	Cerebellum_9_R	LimN	8.967	0.681	[0.293,1.068]	0.003
116	Cerebellum_Crus2_R	FPN	6.927	0.481	[0.097,0.864]	0.009

Effect sizes, p-values, and directional effects comparing ASD-only and ASD+ADHD subgroups in regions meeting statistical thresholds.

To check if these subgroup findings were robust and independent, we looked at these same regions in all our group comparisons (**Table 4**). The Cerebellum_9_R showed no significant effect in ASD-only versus TD (*d* = 0.05, *p* = 0.408) but a large deficit in ADHD versus TD (*d* = -0.68, *p* = 0.001). This means its increased coupling in the ASD+ADHD group likely reflects the incorporation of a trait related to ASD+ADHD. itself. For the ITG.L, it was a significant feature in ASD-only versus TD (*s* = -0.31, *p* = 0.037), but this difference was absent in ASD+ADHD versus TD (*d* = 0.08, *p* = 0.375). Its weaker coupling in the comorbid group therefore suggests that ADHD may specifically attenuate this typical ASD characteristic. Furthermore, the Cerebellum_Crus2_R was not a significantly different region in ASD versus TD (*p* = 0.07), confirming that its alteration is a new finding specific to the subgroups. The medium-to-large effect sizes (Cohen’s *d* from 0.48 to 0.68) with confidence intervals not including zero give us confidence that these are real biological effects, even though the ASD+ADHD group was smaller.

**Table 4:** Cross-comparison profiles of key regions supporting subgroup differences.

Name	Network	Comparison	Cohen’s *d*	95%CI	*p*_adj_
ITG.L	AudN	ASD-only vs. TD	-0.313	[-0.563, -0.063]	0.037
		ASD+ADHD vs. TD	0.078	[-0.266, 0.423]	0.375
		ASD-only vs. ASD+ADHD	-0.437	[-0.819, -0.055]	0.010
Cerebellum_9_R	LimN	ASD-only vs. TD	0.05	[-0.196, 0.302]	0.408
		ASD+ADHD vs. TD	-0.677	[-1.027, -0.327]	0.001
		ASD-only vs. ASD+ADHD	0.681	[0.293,1.068]	0.003
Cerebellum_Crus2_R	FPN	ASD-only vs. TD	0.148	[-0.102, 0.398]	0.07
		ASD+ADHD vs. TD	-0.278	[-0.624,0.068],	0.268
		ASD-only vs. ASD+ADHD	0.481	[0.097,0.864]	0.009

### Clinical correlations of SC-FC coupling

We next examined whether the observed SC-FC coupling differences between ASD subgroups were related to clinical symptoms. Partial correlation analyses were performed within each subgroup, controlling for age, sex, performance IQ, mean framewise displacement, total intracranial volume, eye status, and acquisition site. False discovery rate correction was applied separately for each subgroup to account for multiple comparisons (**Table 5**).

**Table 5:** Correlations between SC-FC coupling and clinical measures in ASD subgroups.

Subgroup	Clinical measure	Brain region	n	*r*	*p*_adj_
ASDonly	ADI-R Social	ITG.L	33	-0.142	0.412
		Cerebellum_9_R		0.308	0.118
		Cerebellum_Crus2_R		0.101	0.369
	SRS Total	ITG.L	34	0.281	0.408
		Cerebellum_9_R		0.183	0.364
		Cerebellum_Crus2_R		0.287	0.363
ASD+ADHD	ADI-R Social	ITG.L	61	-0.044	0.111
		Cerebellum_9_R		-0.083	0.235
		Cerebellum_Crus2_R		0.096	0.118
	SRS Total	ITG.L	21	0.201	0.283
		Cerebellum_9_R		0.691	0.004
		Cerebellum_Crus2_R		0.021	0.513

In the ASD-only group, we found no significant correlations between SC-FC coupling in the three identified regions and either ADI-R social scores or SRS total scores after multiple comparison correction (all *p* > 0.05). This suggests that the neural alterations in ASD-only may not follow a simple linear relationship with core autism symptoms.

In contrast, the ASD+ADHD group showed a distinct pattern. We observed a significant positive correlation between SC-FC coupling in the Cerebellum_9_R and SRS total scores (*p* = 0.004). This indicates that individuals with ASD+ADHD who showed stronger structural-functional coupling in this cerebellar region also exhibited more severe social responsiveness deficits. No other correlations in the ASD+ADHD group reached statistical significance after correction.

These findings provide clinical context for the SC-FC coupling differences identified between subgroups. The specific association between cerebellar coupling and social symptoms in ASD+ADHD suggests that this neural feature may have direct behavioral relevance in the comorbid condition.

### Structural covariance patterns revealed by PCA

**Figure 8:**
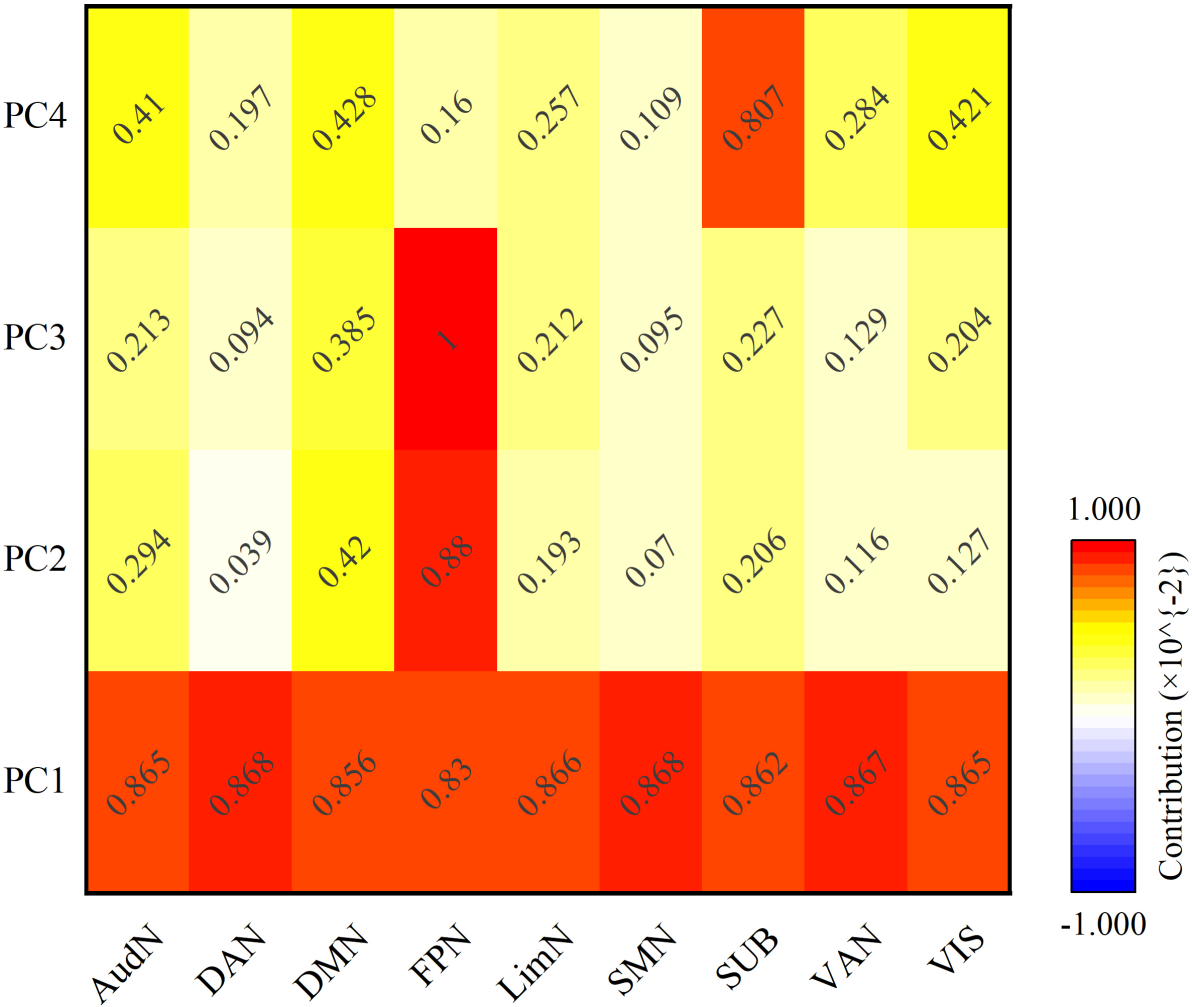
Heatmap of structural network contributions to T1-derived principal components. This visualization maps differential loading patterns of brain networks across covariance components. Color intensity represents each network’s weight in the PCA decomposition of structural features.

**Figure 9:**
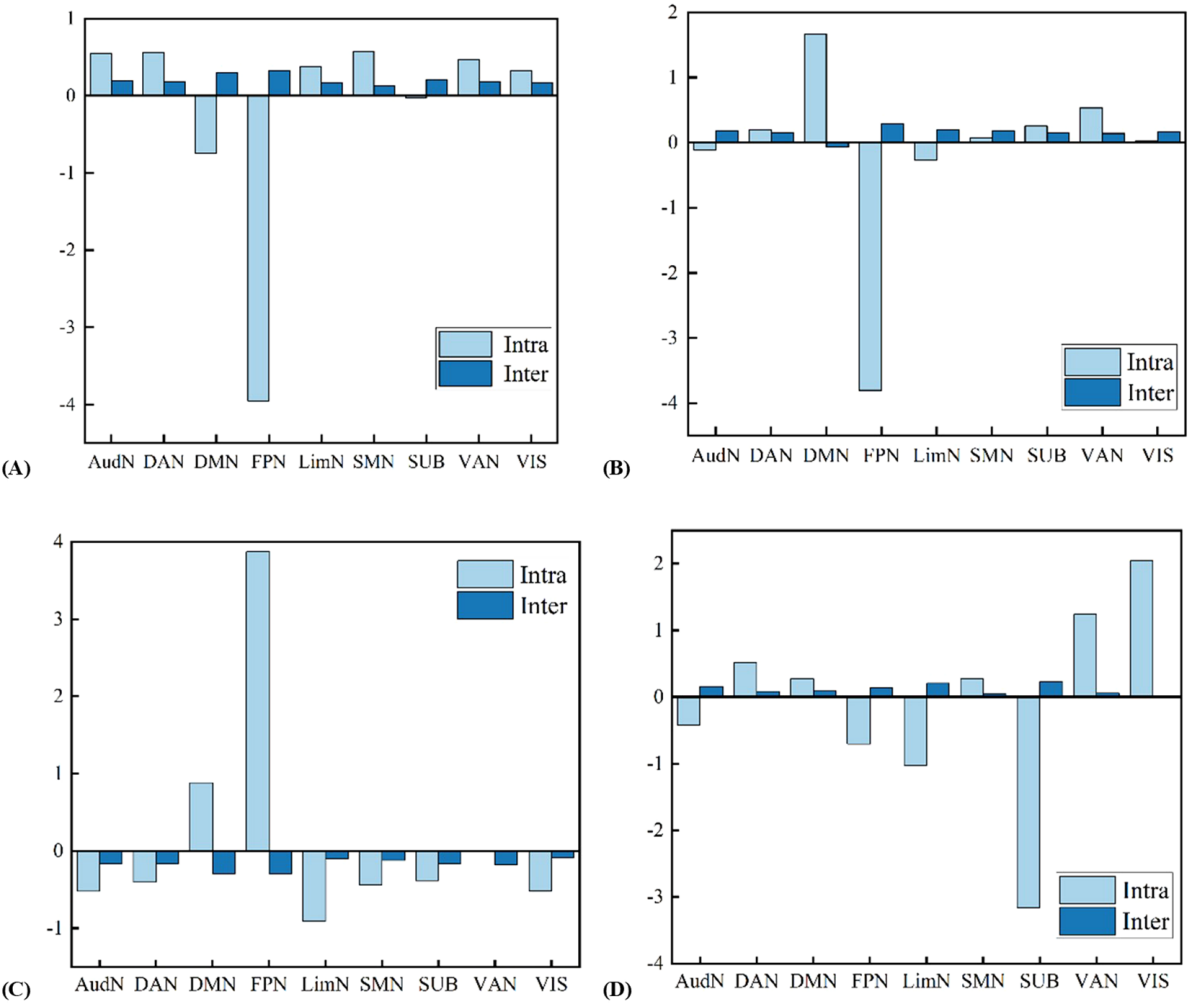
Standardized network strength patterns across principal components. Component-specific alterations in intra-network connectivity strength are shown, highlighting regions with significant enhancement or suppression effects. **(a)** PC1 Z-scored network strength. **(b)** PC2 Z-scored network strength. **(c)** PC3 Z-scored network strength. **(d)** PC4 Z-scored network strength.

**Figure 10:**
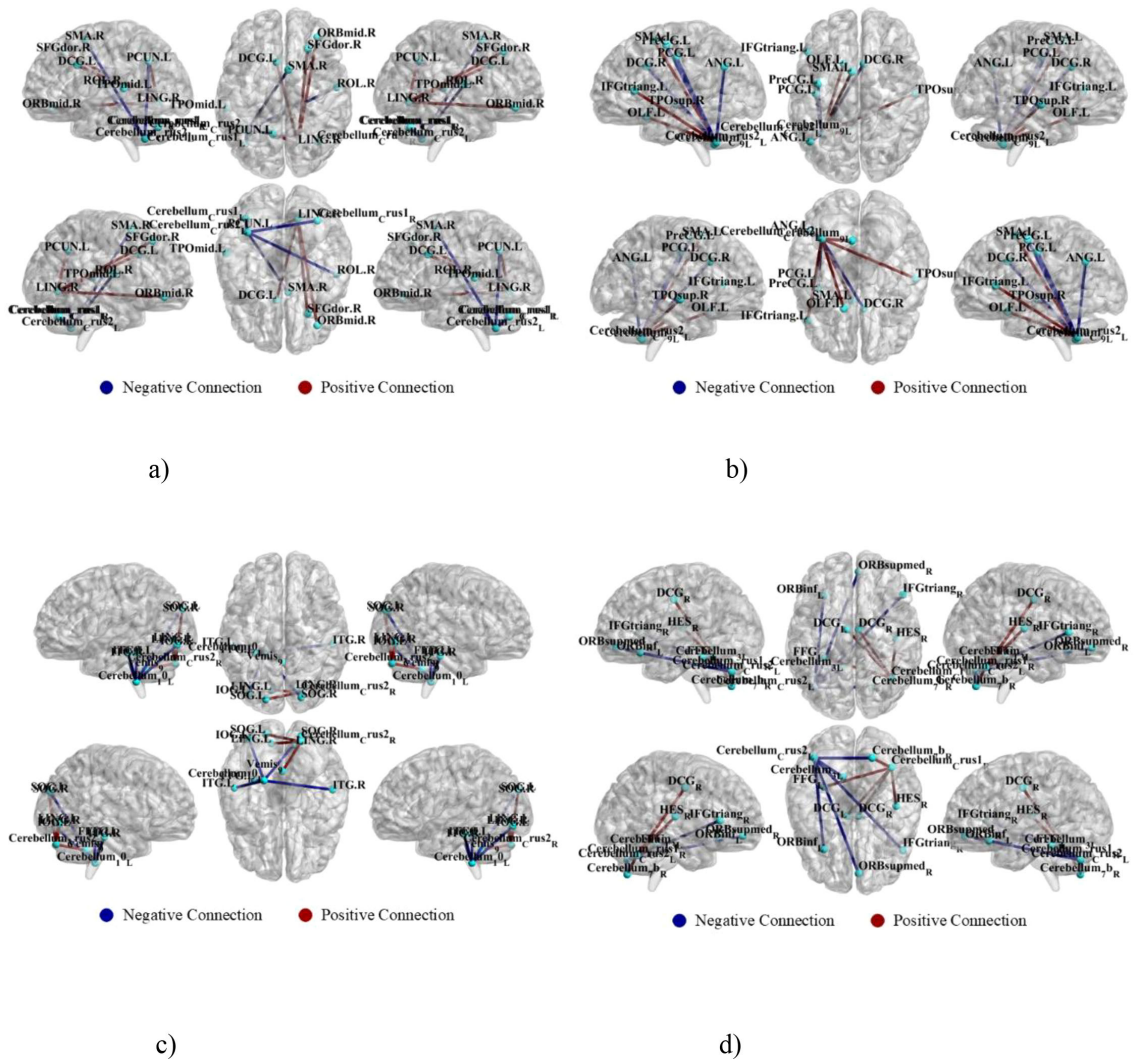
Key subnetworks driving principal component expression. Critical inter-regional pathways contributing to each component’s covariance pattern are identified, showing directional changes in structural connectivity. **(a)** PC1 key subnetwork. **(b)** PC2 key subnetwork. **(c)** PC3 key subnetwork. **(d)** PC4 key subnetwork.

PCA on the structural features produced four components (PCs) that accounted for 99.69% of the variance. We interpreted each PC by identifying its key driving features (**Table 6**). PC1 represented a ‘Global Efficiency’ dimension, driven by short path lengths. PC2 represented an ‘Integration’ dimension. PC3 was a ‘Spatial Constraint’ dimension, linked to Euclidean distance. PC4 was a ‘Modular Hub’ dimension, associated with subgraph centrality. The spatial patterns of these PCs (**Figures 8**–**10**) are the large-scale manifestations of these organizational principles.

**Table 6:** Top driving features based on component loadings for each Principal Component.

Principal component	Top driving feature 1 (loading)	Top driving feature 2 (loading)	Biological interpretation
PC1	Shortest Path (0.999)	Distance (0.998)	Global Efficiency and Cost Dimension
PC2	Distance (0.85)	Shortest Path (0.80)	Integration-Segregation Dimension
PC3	Euclidean (0.90)	–	Physical Spatial Constraint Dimension
PC4	Subgraph Centrality (0.82)	–	Modular Hub Dimension

### Global network architecture

Structural features derived from T1-weighted images underwent PCA, which identified four main patterns of structural covariance. Together, these patterns explain 99.69% of the total variance. Principal component 1 (PC1) alone accounts for 98.64%, PC2 for 0.53%, PC3 for 0.41%, and PC4 for 0.11% (**Figure 8**). These patterns show how brain organization shifts from overall coordination to more specific functions.

PC1 involves widespread activity across nearly the whole brain, driven by the dorsal attention network (DAN). PC2 and PC3 are mainly driven by the frontoparietal network (FPN). PC4 highlights the central role of the subcortical network (SUB).

This component shows widespread increases in connectivity between brain networks. Specifically, connections between hemispheres in the Cerebellum Crus II regions (Cerebellum_Crus2_L and Cerebellum_Crus2_R) are significantly suppressed (**Figure 9a**). At the same time, the DAN pathway linking the right dorsal superior frontal gyrus (SFGdor.R) and the left precuneus (PCUN.L) is strengthened (**Figure 10a**).

PC2 captures abnormal reorganization in the executive control network. FPN disintegration manifests through decreased intra-network strength (**Figure 9b**). Concurrently, compensatory enhancement of connectivity between the right inferior frontal gyrus pars opercularis (IFGoperc.R) and right cerebellum Crus 1 (Cerebellum_Crus1_R) occurred (**Figure 10b**).

PC3 is primarily driven by FPN. A core feature is enhanced intra-network strength in both FPN and DMN (**Figure 9c**). Concurrently, suppressed connectivity between the left middle occipital gyrus (MOG.L) and Cerebellum_9_R occurred (**Figure 10c**).

PC4 characterizes cross-module coordination dominated by subcortical networks. A key alteration is enhanced connectivity from the left cerebellar VIIb (Cerebellum_7b_L) region to left cerebellum Crus I (Cerebellum_Crus1_L) (**Figure 10d**). Simultaneously, significant suppression occurs in connections from the right superior medial orbital frontal cortex (ORBsupmed.R) to Cerebellum_Crus1_R (**Figure 9d**).

### Group differences in covariance patterns

The expression of these components was compared across groups (**Table 7**). Notably, the fundamental coordination pattern of PC1 remained stable across all three participant groups (*F* = 0.337, *p* = 0.714).

**Table 7:** Principal component characteristics and clinical associations.

PC	Explained variance	Dominant network	Clinical significance
PC1	98.64%	DAN	No group difference (*F* = 0.337, *p* = 0.714)
PC2	0.53%	FPN	ASD > TD (*F* = 3.46, *p* = 0.032)
PC3	0.41%	FPN	No group difference (*F* = 1.48, *p* = 0.228)
PC4	0.11%	SUB	No group difference (*F* = 0.349, *p* = 0.705)

Summary of variance contributions, dominant networks, and group difference statistics for all components.

Critically, the expression of PC2 differed across groups: ASD participants showed elevated values (1.96 ± 11.78) versus typically developing controls (-0.83 ± 6.95) (*F* = 3.46, *p* = 0.032), while ASD+ADHD participants (-0.30 ± 5.45) did not differ from controls. This implicates abnormal FPN-cerebellar pathways as a specific neural marker for ASD executive dysfunction.

The visuospatial integration pattern of PC3 revealed no group differences (*F* = 1.48, *p* = 0.228). The cross-module coordination pattern of PC4 also showed no significant group differences (*F* = 0.35, *p* = 0.71).

PC1 explains 98.64% of the variance and reflects a whole-brain coordination mechanism driven by DAN. This component shows widespread increases in connectivity between brain networks. Specifically, connections between hemispheres in the Cerebellum Crus II regions (Cerebellum_Crus2_L and Cerebellum_Crus2_R) are significantly suppressed (**Figure 9a**). At the same time, the DAN pathway linking the right dorsal superior frontal gyrus (SFGdor.R) and the left precuneus (PCUN.L) is strengthened (**Figure 10a**). Notably, this fundamental coordination pattern remained stable across all three participant groups (*F* = 0.337, *p* = 0.714), indicating it is a core feature of the brain’s network architecture.

PC2 explains 0.53% of variance and captures abnormal reorganization in the executive control network. FPN disintegration manifests through decreased intra-network strength (**Figure 9b**). Concurrently, compensatory enhancement of connectivity between the right inferior frontal gyrus pars opercularis (IFGoperc.R) and right cerebellum Crus 1 (Cerebellum_Crus1_R) occurred (**Figure 10b**). Critically, component expression differed across groups: ASD participants showed elevated values (1.96 ± 11.78) versus typically developing controls (-0.83 ± 6.95) (*F* = 3.46, *p* = 0.032), while ADHD participants (-0.30 ± 5.45) did not differ from controls. This implicates abnormal FPN-cerebellar pathways as a specific neural marker for ASD executive dysfunction.

PC3 accounts for 0.41% of variance and is primarily driven by FPN. A core feature is enhanced intra-network strength in both FPN and DMN (**Figure 9c**). Concurrently, suppressed connectivity between the left middle occipital gyrus (MOG.L) and Cerebellum_9_R occurred (**Figure 10c**). This visuospatial integration pattern revealed no group differences (*F* = 1.48, *p* = 0.228), suggesting a conserved sensory processing mechanism across populations.

PC4 accounts for 0.11% of variance and characterizes cross-module coordination dominated by subcortical networks. A key alteration is enhanced connectivity from the left cerebellar VIIb (Cerebellum_7b_L) region to left cerebellum Crus I (Cerebellum_Crus1_L) (**Figure 10d**). Simultaneously, significant suppression occurs in connections from the right superior medial orbital frontal cortex (ORBsupmed.R) to Cerebellum_Crus1_R (**Figure 9d**). This pattern showed no significant group differences (*F* = 0.35, *p* = 0.71).

Together, these four patterns build a layered model of brain network structure. Within this framework, PC1 serves as a basic coordination layer supporting information integration across the entire brain. PC2 acts as an executive control layer for flexible cognitive control, while PC3 serves as a sensory integration layer processing visual and spatial inputs. PC4 operates as a cross-network coordination layer regulating interactions between different neural systems. Importantly, specific cerebellar regions perform distinct functions within this hierarchy: the Crus I lobule contributes to executive control (PC2), lobule VIIb mediates sensory-motor integration (PC3 and PC4), and Crus II maintains global coordination (PC1). This expanded understanding of the cerebellum, moving beyond its traditional motor role, offers new insights into the neural architecture of neurodevelopmental disorders.

### Hemispheric lateralization patterns from PCA

#### Global lateralization architecture

**Figure 11:**
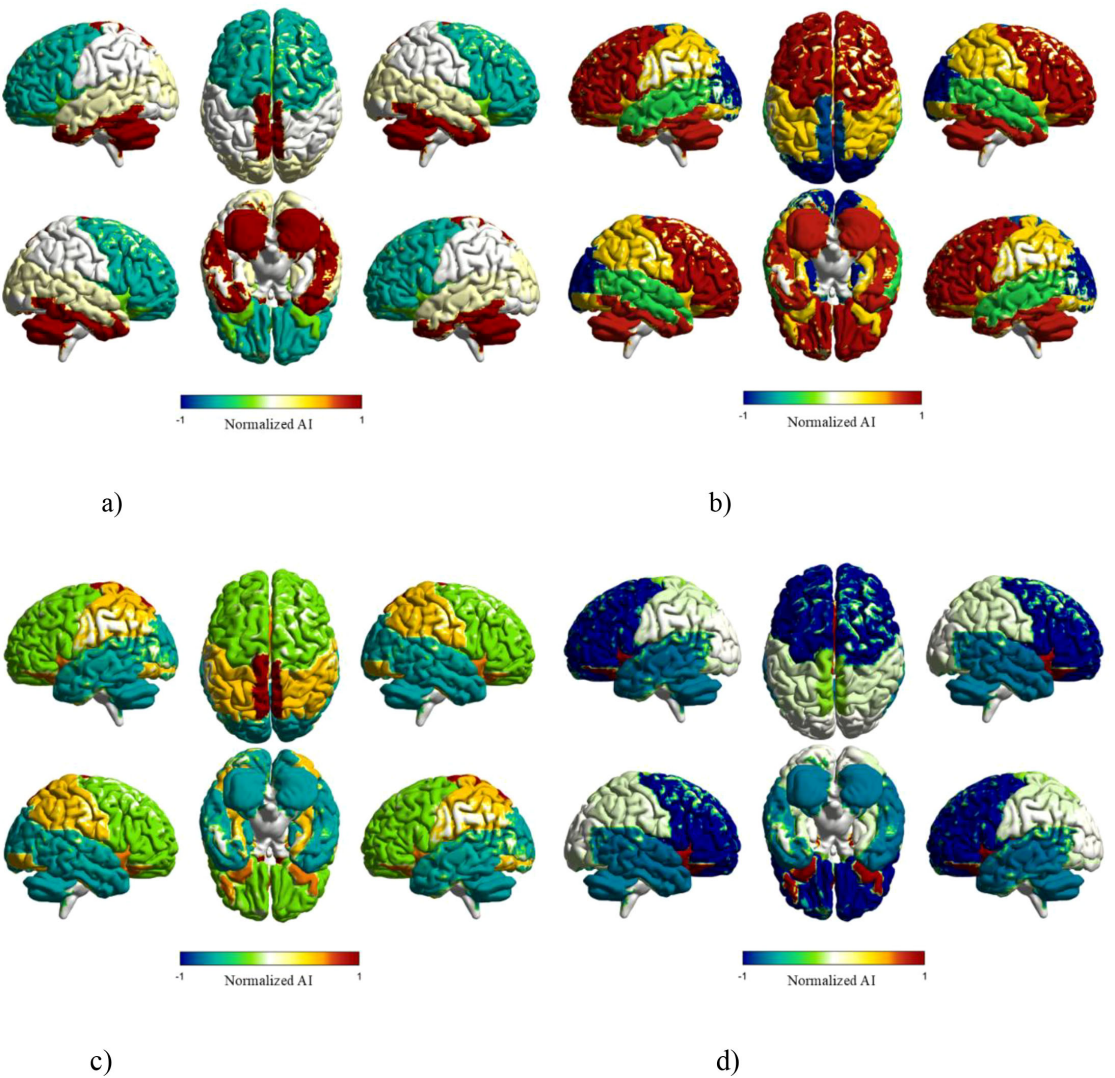
Principal components capture hemispheric asymmetry patterns. Asymmetry differences across seven brain regions are visualized through four principal components. Unique left-right profiles emerge per component, with PC2 exhibiting the strongest contrast: left-leaning occipital cortex versus right-leaning cerebellum and parietal areas. **(a)** PC1 asymmetry index. **(b)** PC2 asymmetry index. **(c)** PC3 asymmetry index. **(d)** PC4 asymmetry index.

We applied PCA to study hemispheric lateralization in brain anatomy. Asymmetry indices (AI) were computed for seven anatomical regions: Frontal lobe, Limbic System, Occipital lobe, Parietal lobe, Subcortical Structures, Temporal lobe, and Cerebellum. The distinct asymmetry profiles captured by the first four principal components are visualized (**Figure 11**).

Specifically, rightward asymmetry is observed in both the cerebellum (AI = 0.0097) and subcortical structures (AI = 3.50) (**Figure 11a**). The strongest left-lateralization occurs in the occipital lobe (AI = -10.35), contrasting with rightward tendencies in both the cerebellum (AI = 3.84) and parietal regions (AI = 0.65) (**Figure 11b**). Right-lateralization is prominent in subcortical structures (AI = 3.50), while temporal regions show a leftward bias (AI = -0.97) (**Figure 11c**). Consistent left-lateralization is present in the frontal lobe (AI = -3.49), complemented by rightward tendencies in limbic areas (**Figure 11d**).

PCA effectively differentiated the lateralization profiles of these functional systems (**Table 8**). Key characteristic patterns included the Occipital lobe’s strong left-bias on the second component, the Frontal lobe’s stable left-lateralization on the fourth component, the Cerebellum’s persistent rightward bias across PC1 and PC2, and the bidirectional shift in Subcortical structures.

**Table 8:** Core brain networks and hemispheric differentiation of principal components.

PC	Core brain region	Functional axis	Lateralization direction	AI	Functional interpretation
PC1	Cerebellum	Sensorimotor Integration	Rightward	0.0097	Motor coordination and error prediction
	Subcortical_Structures	Limbic-Striatal Circuit	Rightward	3.50	Reward processing
PC2	Occipital Lobe	Visual Processing	Leftward	-10.35	Object recognition and detailed analysis
	Parietal Lobe	Spatial Attention	Rightward	0.65	Spatial navigation
	Cerebellum	Motor Sequencing	Rightward	3.84	Action sequence coordination
PC3	Subcortical_Structures	Emotion Regulation	Rightward	3.50	Emotional response processing
	Temporal Lobe	Auditory Processing	Leftward	-0.97	Language comprehension
PC4	Frontal Lobe	Executive Control	Leftward	-3.49	Working memory and decision-making
	Limbic System	Social Cognition	Rightward	2.74	Social interaction processing

Principal components map asymmetric functional organization across core networks, with directional biases and quantitative asymmetry defining specialized neural processing pathways.

PCA effectively differentiated the lateralization profiles of these functional systems (**Table 8**). Key characteristic patterns included the Occipital lobe’s strong left-bias on the second component, the Frontal lobe’s stable left-lateralization on the fourth component, the Cerebellum’s persistent rightward bias across PC1 (AI = 0.0097) and PC2 (AI = 3.84), and the bidirectional shift in Subcortical structures which showed left-lateralization on the second component (AI = -5.04) but switched to right-lateralization on the third component.

#### Group differences in lateralization patterns

**Figure 12:**
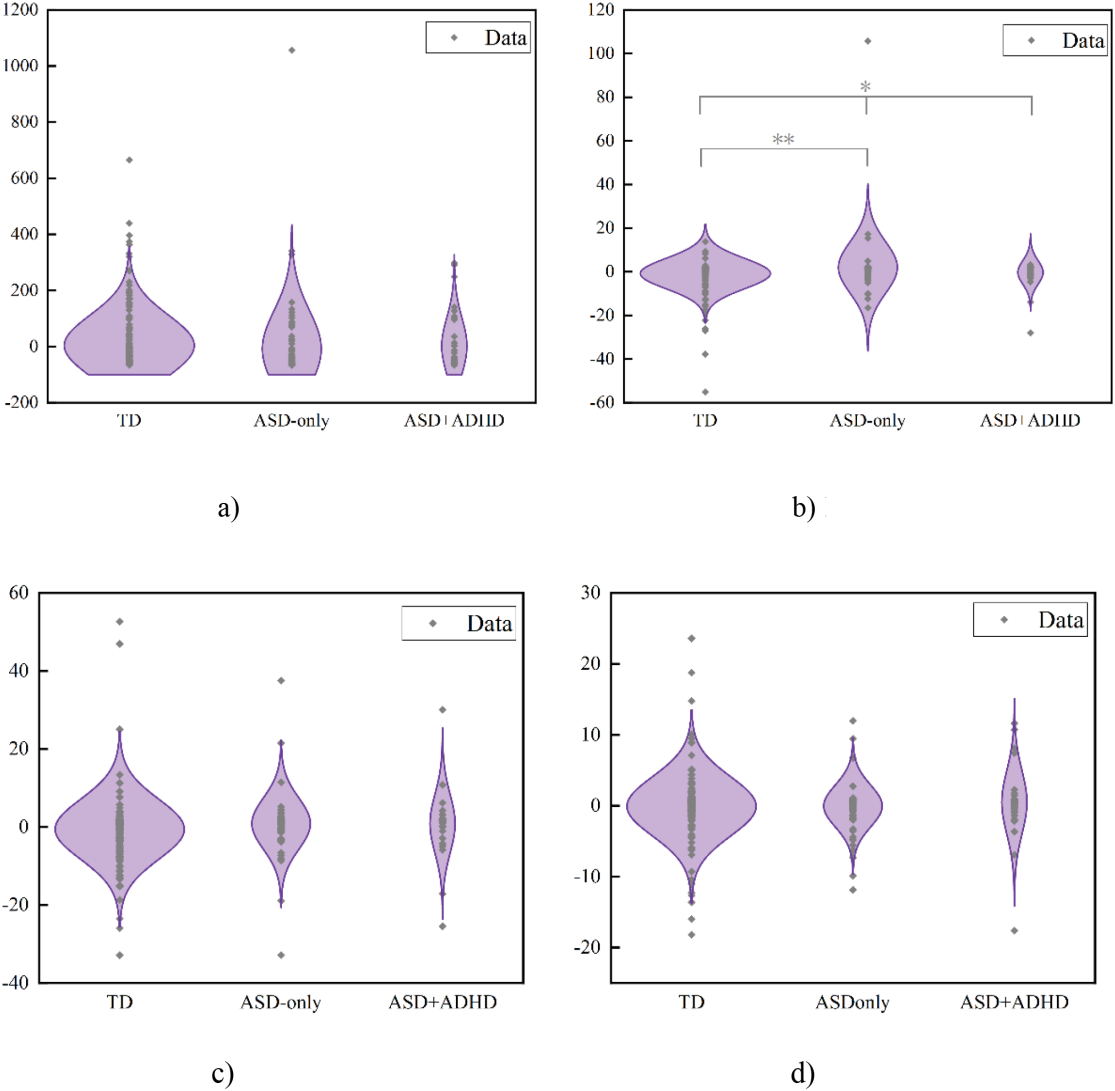
Group differences in principal component scores. Component expression patterns distinguish clinical groups. PC2 separates ASD through distinct occipital-parietal asymmetry, while frontal systems remain stable across diagnoses and the cerebellum shows adaptive adjustments. **(a)** PC1 scores. **(b)** PC2 scores. **(c)** PC3 scores. **(d)** PC4 scores.

The expression of these lateralization components was compared across clinical groups (**Table 9**, **Figure 12**).

**Table 9:** Clinical group differentiation through principal components.

PC	Group	Cohen’s *d*	*p*	Clinical interpretation
PC1	ASD-only vs TD	-0.103	>0.05	Cerebellar functional compensatory trend
	ASD-only vs ASD+ADHD	-0.089	>0.05	No significant intergroup difference
	ASD+ADHD vs TD	-0.013	>0.05	No significant difference
PC2	ASD-only vs TD	0.331	0.009	Occipito-parietal system reorganization
	ASD-only vs ASD+ADHD	0.268	>0.05	Non-significant trend
	ASD+ADHD vs TD	0.063	>0.05	No significant difference
PC3	ASD-only vs TD	0.195	>0.05	Potential alteration in emotional networks
	ASD-only vs ASD+ADHD	0.004	>0.05	No intergroup difference
	ASD+ADHD vs TD	0.191	>0.05	No significant difference
PC4	ASD-only vs TD	0.012	>0.05	Stability of frontal executive networks
	ASD-only vs ASD+ADHD	0.145	>0.05	Negative result
	ASD+ADHD vs TD	-0.133	>0.05	No significant difference

Principal components reveal statistically distinct neural profiles across clinical cohorts, with Cohen’s *d* and *p* linking neural variations to behavioral phenotypes.

Principal components exhibited diagnosis-specific patterns, with only PC2 reaching statistical significance. For PC2, ASD-only exhibited significantly higher values than TD (*t* = 2.062, *p* = 0.009), with a medium effect size (Cohen’s *d* = 0.331) (**Figure 12b**). The observed effect correlated with PC2’s characteristic spatial pattern featuring extreme leftward occipital asymmetry and rightward parietal bias.

While PC3 showed an elevated trend in ASD-only (Cohen’s d = 0.195) (**Figure 12c**), it remained non-significant (p > 0.05). Conversely, the frontal system demonstrated notable cross-group stability. Frontal lobe lateralization patterns on PC4 showed no significant differences among ASD-only, ASD+ADHD, and TD (F = 0.35, p = 0.71) (**Figure 12d**). Cerebellar organization revealed potential compensatory adaptation, with significantly lower PC1 scores in ASD-only inversely related to its baseline rightward asymmetry (Cohen’s d = -0.103) (**Figure 12a**).

These analyses suggest three primary findings. First, diagnosis-specific occipito-parietal alterations in ASD-only individuals were captured by PC2. Second, frontal lateralization patterns represented by PC4 maintained cross-diagnostic stability. Third, cerebellar functional adaptation emerged through PC1. The non-significant trend (*p* > 0.05) toward elevated subcortical rightward asymmetry in ASD-only on PC3 may indicate subtle emotion network modifications. Collectively, the PCA framework delineated multidimensional lateralization features showing differential expression across neurodevelopmental conditions.

## Discussion

ASD is a highly heterogeneous disorder. To address this, we integrated multimodal brain imaging data. Our study provides a systematic map of multi-scale SC-FC coupling abnormalities in the ASD brain. We also identified distinct compensatory pathways in different ASD subtypes. These findings advance our understanding of ASD neurodevelopment.

### Aberrant development of SC-FC coupling in ASD

Our study revealed multi-level disruptions in SC-FC in individuals with ASD. At the macro level, key regions of DMN and LimN showed widespread reductions in SC-FC coupling. Affected areas include the superior temporal gyrus (STG.L/R), posterior cingulate gyrus (PCG.L), and amygdala (AMYG.R). These changes correspond to core ASD deficits in self-referential processing and emotional regulation (9, 47) and align with patterns of abnormal amygdala development observed in ASD (48). Importantly, significant abnormalities in the Cerebellum_9_R within the limbic network challenge traditional views of limbic system anatomy. This suggests cerebellar involvement in socioemotional processing through cortico-cerebellar circuits (49–51).

At the intermediate level, the FPN and VAN displayed a dynamic imbalance. Compensatory increases in coupling at the IFGoperc.R coexist with functional deficits at the left inferior parietal lobule (IPL.L). This pattern reflects impaired adaptive control in attention regulation (52, 53). At the micro level, the cerebellum showed developmental timing differences. Early impairments occur in anterior sensorimotor regions like Vermis_4_5. In contrast, posterior cognitive regions such as Cerebellum_Crus2_R demonstrated compensatory activation. This regional contrast supports theories of cerebellar functional specialization during development (50, 54). It also provides direct evidence for neural compensation mechanisms (55–57).

### Neural compensation and network reorganization in ASD subgroups

Our application of PCA was key to identifying these subgroup differences. By distilling the structural data into core ‘brain network organizational dimensions’, we could move beyond regional analyses. This approach revealed that the neural alterations in ASD are not simply a matter of increased or decreased connectivity strength, but involve distinct disruptions in fundamental network principles. Specifically, we found that ASD-specific alterations were most prominent in the Integration-Segregation dimension (PC2), while the comorbidity with ADHD was characterized by additional changes in cerebellar-related dimensions.

Our study revealed distinct compensatory mechanisms in ASD subgroups. In ASD-only, increased SC-FC coupling in the ITG.L served as a core compensatory pathway. This finding connects directly to the region’s established role in social semantic processing (58–60). Although ADI-R social scores didn’t reach statistical significance (*p* > 0.05), the trend toward higher scores in ASD-only versus comorbid cases suggests ITG.L may buffer social deficits through neural reorganization. This supports the adaptive compensation hypothesis in high-functioning ASD (55, 56).

For ASD+ADHD, characteristic increases in cerebellar cognitive regions (Cerebellum_9_R/Cerebellum_Crus2_R) revealed a unique strategy. This pattern involves shifting attentional control functions to the cerebellum. It confirms the role of cerebello-prefrontal circuits in executive functioning (51, 61). Importantly, it provides an integrated framework explaining comorbid neural mechanisms (62, 63), distinct from the fronto-insular-thalamic dysfunction sometimes seen in pure ADHD (64).

Furthermore, the PCA model itself provided a developmental perspective. The prominence of the Integration-Segregation dimension (PC2) in ASD suggests that the core neuropathology may stem from impaired optimization of executive networks, potentially due to aberrant synaptic pruning (23, 40). This executive network disruption may also be linked to the observed visual processing alterations (65, 66).

### Clinical implications

Our findings suggest several clinically relevant pathways for advancing precision medicine in ASD. Evidence indicates that distinct activation in Cerebellum_Crus2_R could serve as an objective biomarker for differentiating the ASD+ADHD (67), while its regulatory mechanism may inform targeted interventions like transcranial stimulation (57, 68). The link we observed between ITG.L coupling and social function, though not statistically strong, suggests this brain region might help predict how people with ASD-only will manage social situations (26, 55). Furthermore, PCA-derived features such as PC2 strength might provide a novel metric for evaluating treatment efficacy in executive function interventions for adolescents (9, 69). For early risk identification, the observed cerebellar coupling gradients appear promising for infant monitoring approaches (50, 54).

### Limitations

Our study has several limitations to consider. First, we had a relatively small number of participants with ASD+ADHD (only 39), which means our findings for this group need to be confirmed with larger studies (3). Second, while we used the standard AAL116 atlas to define brain regions, this method might miss some fine details in brain connections (69, 70). Future studies could use more detailed brain mapping methods to get a clearer picture (15, 60, 71). Third, we didn’t account for whether participants were taking medication, which could have affected our results, particularly for the ASD+ADHD group (47, 72). Fourth, although we used standard methods to combine data from different scanning sites, small differences in scanning equipment might still have influenced our findings. Finally, we only looked at average brain connections over time, but studying how these connections change moment-to-moment might reveal important patterns we missed (69, 70).

## Conclusions

Our study identified multi-level alterations in brain structure-function relationships in adolescents with ASD. These changes form a hierarchy of disruptions, from shared network-level impairments to distinct, subtype-specific adaptations, against a background of globally reorganized network architecture.

At the group level, adolescents with ASD showed widespread reductions in structure-function coupling, particularly within the default mode and limbic networks. These impairments in regions critical for self-referential and emotional processing are likely linked to the core social communication difficulties in ASD.

We further found that these common changes support divergent neural pathways in different clinical subtypes. In ASD without ADHD, we observed stronger coupling in the left inferior temporal gyrus, which may represent a subtype-specific compensatory mechanism for social semantic processing. In contrast, the ASD+ADHD subtype was characterized by enhanced coupling in cerebellar regions, suggesting a unique reliance on cerebellar circuits for attentional control.

Principal component analysis confirmed that these circuit-level alterations are part of a broader systematic reorganization. We identified a stable whole-brain coordination system, alongside a specifically elevated component reflecting the reorganization of frontoparietal-cerebellar pathways for executive control.

In summary, our results delineate both common impairments and distinct subtype signatures within a hierarchically reorganized brain network in ASD. This multi-level perspective provides a more nuanced framework for understanding ASD heterogeneity and could inform the development of precisely targeted interventions for different clinical presentations.

## Data Availability

Publicly available datasets were analyzed in this study. This data can be found here: The dataset analyzed in this study is publicly available from the Autism Brain Imaging Data Exchange II (ABIDE II) repository hosted on the International Neuroimaging Data-sharing Initiative (INDI) platform at https://fcon_1000.projects.nitrc.org/indi/abide/abide_II.html (Persistent Identifier: DOI 10.15787/VTT1/MD5YH6).

## Supplementary material

Supplementary Table 1 Brainnetome Network Parcellations with Region Codes and Anatomic Labels.

Supplementary Table 2 Brainnetome Atlas Lobar Regions with Numeric Codes and Anatomic Names.
