# Supplementary material for: Structural and functional coupling alterations in autism spectrum disorder with and without comorbid attention deficit hyperactivity disorder
Source: Front Psychiatry. 2026 Jan 15;16:1704170. doi: 10.3389/fpsyt.2025.1704170 (PMC12852334; doi:10.3389/fpsyt.2025.1704170)
Supplement: Supplementary Table 1 — Brainnetome Network Parcellations with Region Codes and Anatomic Labels. [file Table1.docx]

Supplementary Material

# Supplementary Tables

**Supplementary Table 1.** Brainnetome Network Parcellations with Region Codes and Anatomic Labels.

| Network | ID | Name |
| --- | --- | --- |
| AudN | 79 | HES.L |
| AudN | 80 | HES.R |
| AudN | 89 | ITG.L |
| AudN | 90 | ITG.R |
| DAN | 3 | SFGdor.L |
| DAN | 4 | SFGdor.R |
| DAN | 59 | SPG.L |
| DAN | 60 | SPG.R |
| DAN | 95 | Vemis_7 |
| DAN | 105 | Cerebellum_7b_L |
| DAN | 106 | Cerebellum_7b_R |
| DMN | 5 | ORBsup.L |
| DMN | 6 | ORBsup.R |
| DMN | 9 | ORBmid.L |
| DMN | 10 | ORBmid.R |
| DMN | 15 | ORBinf.L |
| DMN | 16 | ORBinf.R |
| DMN | 23 | SFGmed.L |
| DMN | 24 | SFGmed.R |
| DMN | 25 | ORBsupmed.L |
| DMN | 26 | ORBsupmed.R |
| DMN | 33 | DCG.L |
| DMN | 34 | DCG.R |
| DMN | 35 | PCG.L |
| DMN | 36 | PCG.R |
| DMN | 63 | SMG.L |
| DMN | 64 | SMG.R |
| DMN | 65 | ANG.L |
| DMN | 66 | ANG.R |
| DMN | 67 | PCUN.L |
| DMN | 68 | PCUN.R |
| DMN | 81 | STG.L |
| DMN | 82 | STG.R |
| DMN | 83 | TPOsup.L |
| DMN | 84 | TPOsup.R |
| DMN | 113 | Cerebellum_Crus1_L |
| DMN | 114 | Cerebellum_Crus1_R |
| FPN | 7 | MFG.L |
| FPN | 8 | MFG.R |
| FPN | 13 | IFGtriang.L |
| FPN | 14 | IFGtriang.R |
| FPN | 61 | IPL.L |
| FPN | 62 | IPL.R |
| FPN | 115 | Cerebellum_Crus2_L |
| FPN | 116 | Cerebellum_Crus2_R |
| LimN | 21 | OLF.L |
| LimN | 22 | OLF.R |
| LimN | 27 | REC.L |
| LimN | 28 | REC.R |
| LimN | 37 | HIP.L |
| LimN | 38 | HIP.R |
| LimN | 39 | PHG.L |
| LimN | 40 | PHG.R |
| LimN | 41 | AMYG.L |
| LimN | 42 | AMYG.R |
| LimN | 85 | MTG.L |
| LimN | 86 | MTG.R |
| LimN | 97 | Vemis_9 |
| LimN | 109 | Cerebellum_9_L |
| LimN | 110 | Cerebellum_9_R |
| SMN | 1 | PreCG.L |
| SMN | 2 | PreCG.R |
| SMN | 17 | ROL.L |
| SMN | 18 | ROL.R |
| SMN | 19 | SMA.L |
| SMN | 20 | SMA.R |
| SMN | 57 | PoCG.L |
| SMN | 58 | PoCG.R |
| SMN | 69 | PCL.L |
| SMN | 70 | PCL.R |
| SMN | 91 | Vemis_1_2 |
| SMN | 92 | Vemis_3 |
| SMN | 93 | Vemis_4_5 |
| SMN | 94 | Vemis_6 |
| SMN | 99 | Cerebellum_3_L |
| SMN | 100 | Cerebellum_3_R |
| SMN | 101 | Cerebellum_4_5_L |
| SMN | 102 | Cerebellum_4_5_R |
| SMN | 103 | Cerebellum_6_L |
| SMN | 104 | Cerebellum_6_R |
| SUB | 71 | CAU.L |
| SUB | 72 | CAU.R |
| SUB | 73 | PUT.L |
| SUB | 74 | PUT.R |
| SUB | 75 | PAL.L |
| SUB | 76 | PAL.R |
| SUB | 77 | THA.L |
| SUB | 78 | THA.R |
| VAN | 11 | IFGoperc.L |
| VAN | 12 | IFGoperc.R |
| VAN | 29 | INS.L |
| VAN | 30 | INS.R |
| VAN | 31 | ACG.L |
| VAN | 32 | ACG.R |
| VAN | 96 | Vemis_8 |
| VAN | 107 | Cerebellum_8_L |
| VAN | 108 | Cerebellum_8_R |
| VIS | 43 | CAL.L |
| VIS | 44 | CAL.R |
| VIS | 45 | CUN.L |
| VIS | 46 | CUN.R |
| VIS | 47 | LING.L |
| VIS | 48 | LING.R |
| VIS | 49 | SOG.L |
| VIS | 50 | SOG.R |
| VIS | 51 | MOG.L |
| VIS | 52 | MOG.R |
| VIS | 53 | IOG.L |
| VIS | 54 | IOG.R |
| VIS | 55 | FFG.L |
| VIS | 56 | FFG.R |
| VIS | 87 | TPOmid.L |
| VIS | 88 | TPOmid.R |
| VIS | 98 | Vemis_10 |
| VIS | 111 | Cerebellum_10_L |
| VIS | 112 | Cerebellum_10_R |

**Supplementary Table 2.** Brainnetome Atlas Lobar Regions with Numeric Codes and Anatomic Names.

| Lobe | ID | Name |
| --- | --- | --- |
| Frontal | 1 | PreCG.L |
|  | 2 | PreCG.R |
|  | 3 | SFGdor.L |
|  | 4 | SFGdor.R |
|  | 5 | ORBsup.L |
|  | 6 | ORBsup.R |
|  | 7 | MFG.L |
|  | 8 | MFG.R |
|  | 9 | ORBmid.L |
|  | 10 | ORBmid.R |
|  | 11 | IFGoperc.L |
|  | 12 | IFGoperc.R |
|  | 13 | IFGtriang.L |
|  | 14 | IFGtriang.R |
|  | 15 | ORBinf.L |
|  | 16 | ORBinf.R |
|  | 17 | ROL.L |
|  | 18 | ROL.R |
|  | 19 | SMA.L |
|  | 20 | SMA.R |
|  | 21 | OLF.L |
|  | 22 | OLF.R |
|  | 23 | SFGmed.L |
|  | 24 | SFGmed.R |
|  | 25 | ORBsupmed.L |
|  | 26 | ORBsupmed.R |
|  | 27 | REC.L |
|  | 28 | REC.R |
| Limbic System | 29 | INS.L |
|  | 30 | INS.R |
|  | 31 | ACG.L |
|  | 32 | ACG.R |
|  | 33 | DCG.L |
|  | 34 | DCG.R |
|  | 35 | PCG.L |
|  | 36 | PCG.R |
|  | 37 | HIP.L |
|  | 38 | HIP.R |
|  | 39 | PHG.L |
|  | 40 | PHG.R |
|  | 41 | AMYG.L |
|  | 42 | AMYG.R |
| Occipital | 43 | CAL.L |
|  | 44 | CAL.R |
|  | 45 | CUN.L |
|  | 46 | CUN.R |
|  | 47 | LING.L |
|  | 48 | LING.R |
|  | 49 | SOG.L |
|  | 50 | SOG.R |
|  | 51 | MOG.L |
|  | 52 | MOG.R |
|  | 53 | IOG.L |
|  | 54 | IOG.R |
|  | 55 | FFG.L |
|  | 56 | FFG.R |
| Parietal | 57 | PoCG.L |
|  | 58 | PoCG.R |
|  | 59 | SPG.L |
|  | 60 | SPG.R |
|  | 61 | IPL.L |
|  | 62 | IPL.R |
|  | 63 | SMG.L |
|  | 64 | SMG.R |
|  | 65 | ANG.L |
|  | 66 | ANG.R |
|  | 67 | PCUN.L |
|  | 68 | PCUN.R |
|  | 69 | PCL.L |
|  | 70 | PCL.R |
| Subcortical Structures | 71 | CAU.L |
|  | 72 | CAU.R |
|  | 73 | PUT.L |
|  | 74 | PUT.R |
|  | 75 | PAL.L |
|  | 76 | PAL.R |
|  | 77 | THA.L |
|  | 78 | THA.R |
| Temporal | 79 | HES.L |
|  | 80 | HES.R |
|  | 81 | STG.L |
|  | 82 | STG.R |
|  | 83 | TPOsup.L |
|  | 84 | TPOsup.R |
|  | 85 | MTG.L |
|  | 86 | MTG.R |
|  | 87 | TPOmid.L |
|  | 88 | TPOmid.R |
|  | 89 | ITG.L |
|  | 90 | ITG.R |
| Cerebellum | 91 | Vemis_1_2 |
|  | 92 | Vemis_3 |
|  | 93 | Vemis_4_5 |
|  | 94 | Vemis_6 |
|  | 95 | Vemis_7 |
|  | 96 | Vemis_8 |
|  | 97 | Vemis_9 |
|  | 98 | Vemis_10 |
|  | 99 | cerebelum_3_L |
|  | 100 | cerebelum_3_R |
|  | 101 | cerebelum_4_5_L |
|  | 102 | cerebelum_4_5_R |
|  | 103 | cerebelum_6_L |
|  | 104 | cerebelum_6_R |
|  | 105 | cerebelum_7b_L |
|  | 106 | cerebelum_7b_R |
|  | 107 | cerebelum_8_L |
|  | 108 | cerebelum_8_R |
|  | 109 | cerebelum_9_L |
|  | 110 | cerebelum_9_R |
|  | 111 | cerebelum_10_L |
|  | 112 | cerebelum_10_R |
|  | 113 | cerebelum_Crus1_L |
|  | 114 | cerebelum_Crus1_R |
|  | 115 | cerebelum_Crus2_L |
|  | 116 | cerebelum_Crus2_R |
